# Associations of ApoE4 status and DHA supplementation on plasma and CSF lipid profiles and entorhinal cortex thickness

**DOI:** 10.1016/j.jlr.2023.100354

**Published:** 2023-03-22

**Authors:** Mikaila Ann Bantugan, Haotian Xian, Victoria Solomon, Mitchell Lee, Zhiheng Cai, Shaowei Wang, Marlon V. Duro, Bilal E. Kerman, Alfred Fonteh, Cristiana Meuret, Meitong Li, Meredith N. Braskie, Laura Beth J. McIntire, Lucia Jurin, Sarah Oberlin, James Evans, Roderick Davis, Wendy J. Mack, Laila Abdullah, Hussein N. Yassine

**Affiliations:** 1 Department of Medicine and Neurology, Keck School of Medicine, University of Southern California, Los Angeles, CA, USA; 2 Department of Neurosciences, Huntington Medical Research Institute, Pasadena, CA, USA; 3 Department of Radiology, Brain Health Imaging Institute, Weill Cornell Medical College, New York City, NY, USA; 4 Roskamp Institute, Sarasota, FL, USA; 5 Department of Population and Public Health Sciences, Keck School of Medicine, University of Southern California, Los Angeles, CA, USA; 6 James A. Haley VA Hospital, Tampa, FL, USA

**Keywords:** ApoE, Lipids, Brain, Alzheimer’s, Lipidomics

## Abstract

Apolipoprotein ε allele 4 (*APOE4*) influences the metabolism of polyunsaturated fatty acids (PUFAs) such as docosahexaenoic acid (DHA). The entorhinal cortex (EC) in the brain is affected early in Alzheimer's disease and is rich in DHA. The purpose of this study is to identify the effect of *APOE4* and DHA lipid species on the EC. Plasma and cerebrospinal fluid (CSF) lipidomic measurements were obtained from the DHA Brain Delivery Pilot, a randomized clinical trial of DHA supplementation (n = 10) versus placebo (n = 12) for six months in nondemented older adults stratified by *APOE4* status. Wild-type C57B6/J mice were fed a high or low DHA diet for 6 months followed by plasma and brain lipidomic analysis. Levels of phosphatidylcholine DHA (PC 38:6) and cholesterol ester DHA (CE 22:6) had the largest increases in CSF following supplementation (*P* < 0.001). DHA within triglyceride (TG) lipids in CSF strongly correlated with corresponding plasma TG lipids, and differed by *APOE4*, with carriers having a lower increase than noncarriers. Changes in plasma PC DHA had the strongest association with changes in EC thickness in millimeters, independent of *APOE4* status (*P* = 0.007). In mice, a high DHA diet increased PUFAs within brain lipids. Our findings demonstrate an exchange of DHA at the CSF-blood barrier and into the brain within all lipid species with *APOE* having the strongest effect on DHA-containing TGs. The correlation of PC DHA with EC suggests a functional consequence of DHA accretion in high density lipoprotein for the brain.

Carrying an apolipoprotein ε allele 4 (*APOE4*) is the strongest genetic risk factor for late-onset Alzheimer’s disease (AD). The ApoE protein plays a role in neuroplasticity and aging and in the exchange of lipids among brain cells. Detrimental alterations in the metabolism of lipids by *APOE4* are one of the possible mechanisms for the *APOE4* genotype-associated increase in AD risk. Among its effects on lipids, ApoE4 affects the metabolism of omega-3 (n-3) and omega-6 (n-6) polyunsaturated fatty acids (PUFAs) (1, 2, 3, 4). An increase in the n-3 docosahexaenoic acid (DHA) is associated with a decrease in the n-6 arachidonic acid (ARA) containing lipids, and this greater ratio is associated with lower measures of neuroinflammation in postmortem brain tissues (5). This occurs because n-3 and n-6 fatty acids compete at the sn-2 position within phospholipids (PLs) for calcium-dependent phospholipase A2 activity (6). *APOE4* is associated with a lower DHA:ARA ratio in both plasma (1) and cerebrospinal fluid (CSF) (4). This likely results from the greater activation of calcium-dependent phospholipase A2 (7) driving a phenotype of chronic unresolved neuroinflammation that associates with lower cognitive functions in *APOE4* dementia (5).

There is an interest in understanding whether n-3 PUFA supplementation can prevent neuroinflammation and its mechanisms of transport into the brain. In the circulation, PUFAs can be esterified to PLs, including phosphatidylcholines (PCs), triglycerides (TGs), and cholesteryl esters (CEs), or transported in free unesterified forms. Among lipoprotein particles, high density lipoprotein (HDL) exhibits a higher proportion of PUFAs within associated PC species compared to low density lipoprotein particles (8). TG-associated PUFAs seem to have a protective role in AD. Lipidomic analysis of plasma samples from the Alzheimer’s Disease Neuroimaging Initiative cohorts identified a correlation between greater plasma TG PUFAs levels and better AD biomarker outcomes such as amyloid β-peptide 42 (Aβ42) levels in CSF or entorhinal cortex (EC) thickness (9). The EC is rich in DHA, and abnormal or deficient EC neural activity is implicated early in late-onset AD pathogenesis. DHA supplementation appears to restore the electrophysiology of EC neurons improving cognitive behaviors such as object recognition (10). Physiologically, the EC feeds into the hippocampus. When the EC functions abnormally, the hippocampus is negatively impacted physiologically and functionally, thus impairing memory performance (11). Therefore, it is plausible that the EC thickness can be used as a surrogate for DHA supplementation brain efficiency.

The preferred lipid species that facilitates the brain delivery and accretion of DHA with aging is not clear. Small HDL particles may play a role in mediating the transport of lipids across the blood-brain barrier (BBB) (12). Several mechanisms are likely involved that include transcytosis of ApoA-1 and its lipidation by the ATP binding cassette 1 (12). In animal models, lysophosphatidylcholine (LPC)-DHA crosses the BBB more efficiently than free DHA due to an interaction with the major facilitator superfamily domain containing 2A (mfsd2a) transporter at the BBB (13). While LPC-DHA may have a greater role in enriching the brain DHA accretion during brain development (14), the preferred DHA lipid pool supplying the aging human brain is not clear.

In the Brain DHA Pilot Delivery trial, we found that high-dose TG-based algal DHA supplementation significantly increased CSF DHA levels and that *APOE4* was associated with lower brain DHA delivery in persons at risk of dementia but without mild cognitive impairment or dementia (2). This study aimed to (1) dissect the 6-months trial changes in lipids measured in CSF and plasma; (2) determine the effect of *APOE4* on DHA-containing lipids; (3) identify the lipid species that best correlate with changes in EC thickness, and (4) test the ability of supplemental DHA to remodel plasma and brain PUFA-containing lipids in wild-type C57B6/J mice.

## MATERIALS AND METHODS

### Participants

Between 2016 and 2018, participants were recruited from the Los Angeles area. We included 22 cognitively unimpaired men and women aged 55 years and older who speak English or Spanish fluently and had a first-degree family history of dementia. Participant demographics are summarized in Table 1. The full description of this cohort has been previously described (2). A clinical dementia rating score (CDR) of greater than 0.5 was used to exclude dementia. The study was approved by the USC IRB (HS-14-00,864) and abides by the declaration of Helsinki. The trial was registered with clinical trials.gov (NCT02541929).

**Table 1:** Demographic and baseline clinical characteristics by treatment arm

Variable	N	Placebo, N = 10	DHA, N = 12	*P*^a^
Age (years), Median (IQR)	22	69.54 (63.36, 70.87)	68.10 (65.97, 71.16)	0.92
Gender, n / N (%)	22			0.57
Female		8 / 10 (80%)	11 / 12 (92%)	
Male		2 / 10 (20%)	1 / 12 (8.3%)	
Race/ethnicity, n / N (%)	22			0.52
Asian		1 / 10 (10%)	0 / 12 (0%)	
Non-Hispanic White		4 / 10 (40%)	7 / 12 (58%)	
Non-Hispanic Black		0 / 10 (0%)	1 / 12 (8.3%)	
Hispanic		4 / 10 (40%)	4 / 12 (33%)	
Other		1 / 10 (10%)	0 / 12 (0%)	
Education (years), Median (IQR)	22	17.00 (14.25, 18.00)	16.50 (16.00, 17.25)	0.89
BMI (kg/m^2^), Median (IQR)	22	32.77 (31.00, 35.06)	26.52 (24.54, 29.26)	0.007
CDR, n / N (%)	22			>0.99
0		10 / 10 (100%)	11 / 12 (92%)	
0.5		0 / 10 (0%)	1 / 12 (8.3%)	
*APOE*, n / N (%)	22			>0.99
*APOE4* noncarriers (2/2, two-thirds, 3/3)		5 / 10 (50%)	5 / 12 (42%)	
*APOE4* carriers (three-fourths, 4/4)		5 / 10 (50%)	7 / 12 (58%)	

CDR, clinical dementia rating; DHA, n-3 docosahexaenoic acid.

a Wilcoxon rank sum exact test; Fisher's exact test; Wilcoxon rank sum test

### Intervention

Participants were randomized to treatment groups of either 2,152 g per day of oral algal DHA or placebo and treated for 6 months in a single-center double-blind trial. Participants were required to consume four 1000 mg soft-gel capsules each day containing either 538 mg DHA (treatment arm) or identical-appearing capsules containing corn/soy oil (placebo arm) manufactured and provided by DSM, Columbia, MD. The capsules had an eicosapentaenoic acid (EPA) level of <0.1% percent of the overall fatty acid makeup. Participants received written instructions to minimize their PUFA intake and were provided and instructed to take two vitamin B complex supplements per day, each comprising 500 µg of vitamin B12, 400 µg of folic acid, and 50 mg of vitamin B6 (Homocysteine Modulators, Solgar, NY). CSF and plasma were collected in polypropylene tubes after overnight fasting. Compliance was determined by pill counting.

### CSF HDL isolation

CSF HDL was isolated via a multistep, density-adjustment flotation ultracentrifugation approach as previously described in Koudinov *et al*. with modifications (15). Briefly, KBr was added to 1 ml of CSF (ρfinal = 1.006 g/ml), then ultracentrifuged at 216,688 *g* for 20 h in a Beckman TL-100 Ultracentrifuge. The top 1/10th volume was collected, and the infranatant’s density was adjusted to 1.063 g/ml, then centrifuged under the same parameters. The top 1/10th layer was collected, and the infranatant’s density was adjusted to 1.25 g/ml, then centrifuged for 48 h before collecting the HDL in the top 1/10th layer. Fractions were dialyzed against a 5 mM NH4OAc buffer (pH = 7.4) at 4°C overnight. Samples were preserved in a 25% sucrose solution and stored at – 80°C prior to mass spectrometry (MS) analysis.

### Transport of supplemental DHA into mouse brain

Twenty-five C57BL/6J mice at 3 months of age, balanced by sex, were subjected to a high DHA diet (open standard diet with 15 kcal% Fat, 1.7 g/kg alpha-linolenic acid, and 7 g/kg DHA, n = 12) or low DHA diet (open standard diet with 15 kcal% fat and 0.9 alpha-linolenic acid per kg, n = 13) for 6 months before sacrifice. The left brain hemisphere was isolated and utilized for lipidomic analyses. However, unlike the clinical trial where DHA supplements that had no n-6 docosapentaenoic acid (DPA) and 60% DHA (supplemental Table S1A), the high DHA diet in the mouse study contained 19% n-6 DPA and 43% DHA (supplemental Table S1B).

### Lipidomics

Except for LPC-DHA, lipidomics measurements were conducted at the Roskamp Institute.

Experimenters blinded to participants’ group membership, and other group characteristics performed sample preparation, lipid extraction, and qualitative quantitative nano-liquid chromatography/MS (LC/MS) analyses on the plasma and CSF from this cohort. Our laboratory previously reported the lipid extraction technique and nano-LC/MS conditions (16). Plasma (10 μl) was spiked with 5 μl of a mix of SPLASH LipidoMIX stable isotope and Cer/Sph Mixture I (Avanti, Polar Lipids, Inc.) diluted 1:10 in SPLASH internal standard (IS) mix. All solvents were HPLC grade purchased from ThermoFisher Scientific. The samples were extracted using a modified Folch method. Methanol (80 μl) was added to the samples, then vortexed for 1 min before adding 120 μl of chloroform and vortexed again for 1 min. Samples were then centrifuged at 4°C at 20,000 relative centrifugal force for 10 min, 40 μl of 0.88% potassium chloride was added to the supernatant in a low-retention Eppendorf microtube and vortexed for 1 min. Samples were centrifuged as before, and the lower phase was transferred to another low retention Eppendorf microtube and evaporated by vacuum centrifugation. For the cleanup procedure, nonsterile microcentrifugal filters (ThermoFisher Scientific) were prepared by applying 200 μl of 1:1 chloroform:methanol to the filters and centrifuging at 4°C, 10,000 relative centrifugal force for 5 min. The flow-through was discarded, and 1:1 chloroform:methanol was added to the samples which were vortexed and then applied to the filters and centrifuged as before. The filters were then discarded and the flow-through was transferred to auto-sampler vials with inserts and dried under vacuum and re-suspended in 50 μl of 70:30 mobile phase A–B, with mobile phase A consisting of 27% isopropanol, 42% water, 31% acetonitrile, 10 mM ammonium formate with the addition of 0.1% formic acid and 90% isopropanol, 10% acetonitrile, 10 mM ammonium formate, and 0.1% formic acid made up mobile phase B.

An Easy-nanoLC 1000 instrument, a nanoflex ESI source, and a Thermo QE/Orbitrap mass spectrometer were utilized. Samples were injected into an Acclaim PepMap 100, 75 μm × 2 cm nanoViper C18, 3 μm, 100 Å trapping column, and Acclaim PepMap RSLC, 75 μm × 15 cm nanoViper C18, 2 μm, 100 Å analytical column for lipid chromatographic separation, running the following gradient at a constant flow rate of 250 nl/min. The starting conditions were 30% B, then from 1 to 50-min program from 50% to 98% B, then switched to 30% B from 50 to 65-min. All samples were run in triplicate in batches of eight along with a blank and quality control (QC) sample. Full-scan MS data were acquired in both positive and negative ion modes, with a mass range of m/z 130–2,000 in the positive ion mode, and m/z 220–2,000 in the negative ion mode, at a resolution of 30,000 for both. The heated capillary was maintained at 200°C, with a spray voltage of 1,500 V. A maximum inject time of 200 ms was used with 13 microscans/acquired scans. Peak areas were integrated using the Tracefinder™ software using a target compound list of lipids of interest containing m/z and retention time for each target lipid and IS for that specific lipid class. For each lipid class, the concentration of lipid species was calculated using the spiked IS corresponding to that class, by dividing the target compound area by the IS area and multiplying by the known IS concentration spiked in. Each species in a sample run that had a coefficient of variance > 25% was excluded from further analysis as considered not to have been measured reliably. Each analytical batch was normalized using its QC ([sample] × [batch QC/normalizing QC]).

### HDL particle analyses

A pool of HDL particle samples was combined to create a combined sample that was used for the Lipid Match workflow to account for possible batch effects. HDL samples (100 μl) were used for lipid extractions from the remaining sample portions as well as the two combined samples were extracted using a Folch procedure using glass vessels. Solvents were added using glass Pasteur pipettes or glass syringes. Before extraction, a 5 μl aliquot of a custom lipid IS mixture was spiked into each sample.

### HDL lipidomics methods

Lipid extracts were analyzed by LC/MS on a system comprised of a Vanquish UHPLC (Thermo Scientific) unit coupled to a Q-Exactive (Thermo Scientific) mass spectrometer through a heated electrospray ionization source. Solvent A was composed of water: acetonitrile (60:40) with 10 mM ammonium acetate and 0.1% acetic acid. Solvent B was composed of 2-propanol: acetonitrile: water (89.02% : 9.89% : 1.09%) with the same modifiers. Separation was carried out on an Acquity UPLC BEH C18 column (Waters, 1.7 um, 1.0 X 50 mm ID) with gradient elution.

Two microliter sample aliquots were loaded onto the column for two minutes with a flow rate of 0.1 ml/min and 10% B. For elution, the gradient was 10%–30% B over 3 min, 30%–45% B over 1 min, 45%–60% B over 2 min, 60%–65% B over 2 min, a hold at 65% B for 2 min, 65%–80% B over 7 min, a hold at 80% B for 1.5 min followed by a return to 10% B over 0.5 min. The flow rate during gradient elution was 0.125 ml/min.

Full scan spectra (m/z 220–2000) with polarity switching were acquired throughout the analysis with a resolving power of 70,000. Automatic gain control target value was set to 3,000,000, the maximum ion trap time was set to 256 ms, and the data type in both modes was centroid.

Quantification of individual lipid species was performed using LipidMatch software or the Tracefinder (Thermo) application as applicable.

### Identification of fatty acid composition of individual lipid species

In order to characterize individual fatty acyl chains, we pooled pretreatment and posttreatment across all samples to generate a representative sample across all samples and subjected it further to LC/MS/MS analyses as described above. TGs and PLs were detected by their MS/MS spectra using their corresponding [M+NH4]^+^ and [M-H]^-^ or [M+CH3COO]^-^, respectively. Based on the assigned exact mass from these MS/MS analyses and their retention times, those compositions are assigned to peaks detected in positive/negative ions using full-scan MS runs. Representative chromatograms are provided in supplemental Fig. S1.

### Measurement of LPC-DHA

Measurement of LPC DHA (LPC 22:6) was done at Huntington Medical Research Institute. Plasma lipids were extracted using a modified Bligh and Dyer method using a Gerstel Robotic workstation. LPC was isolated using hydrophilic interaction chromatography, and the parent ion scans of 184 corresponding to the phosphocholine headgroup were quantified using electrospray ionization liquid chromatography/tandem mass spectrometry (LC/MS-MS). For each sample, the m/z corresponding to LPC species with different fatty acyl chain lengths were extracted and analyzed (486, LPC O-16:0 d_4_; 496, LPC 16:0; 518, LPC 18:3; 520, LPC 18:2; 522. LPC 18:1, 524, LPC 18:0; 542, LPC 20:5; 544, LPC 20:4; 546, LPC 20:3; 548, LPC 20:2; 550, LPC 20:1; 552, LPC 20:0; 568, LPC 22:6; 570, LPC 22:5; 572, LPC 22:4; 574, LPC 22:3; 576, LPC 22:2; 578, LPC 22:1; 580, LPC 22:0) using the LC-Quant module of the XCalibur software (Thermo Scientific, San Jose, CA). LPC containing DHA was expressed as a percentage of the total LPC in each sample. Quantification of individual lipid species was performed using LipidMatch software or the Tracefinder (Thermo) application.

### Determination of EC thickness

We acquired whole-brain magnetization and prepared–rapid gradient echo T1-weighted brain images (3T Siemens Prisma) at baseline and the after the 6-months treatment period. Cortical thickness in the left and right EC was evaluated using FreeSurfer v5.3.0, and segmentations were visually quality checked using a standard protocol. In addition, mean bilateral EC thickness was calculated and used in the analyses.

### Statistical analysis

Baseline participant characteristics were compared between randomized groups using Fisher’s exact test and Wilcoxon rank-sum test. Changes in fatty acids (6-months follow-up minus baseline) as a function of treatment and *APOE4* genotype were modeled using an ANCOVA model within a general linear model framework. The model covariates were baseline lipid measurements and either *APOE4* carrier status or treatment status. An interaction term of treatment status times *APOE4* carrier status was added to the model. Model residuals were evaluated for normality and homoscedasticity. The network graphs were generated using a Pearson’s correlation matrix constructed from the change of different types of lipids, including CEs, ceramides, diacylglycerol, PCs, sphingomyelin, and TGs. The different subspecies of lipids (e.g., PC(30:0), PC(30:1), et cetera) were then filtered for those with statistically significant changes posttreatment using the ANCOVA models described above. In total, 252 such subspecies of lipids in CSF and plasma were evaluated before filtering, and 64 subspecies of lipids remained after filtering. Next, a Pearson’s correlation matrix was calculated for the correlations between those lipids. The matrix was then used to generate a weighted correlation network with a force-embedded layout, which uses an iterative algorithm that makes the nodes in an initial circle layout either repulse or attract each other at each iteration based on their weights. The weights in the network were Pearson’s correlation coefficients between the lipids.

For both plasma and brain mice data analyses, the comparison of the concentrations of each lipid between high DHA and low DHA groups was obtained using an independent *t* test, and the ratio between the mean concentrations of the high group and low groups was calculated. DHA, ARA, EPA, and DPA lipids were selected and used to explore the relationship between brain and plasma lipids. The beta estimates were obtained using generalized linear models between lipids in brain and plasma with the DHA treatment group as the covariate. The standardized beta estimates were obtained by dividing the corresponding lipid by the SD of that lipid in the total group.

In the analyses of the correlations between EC thickness and lipid pools, linear models were run using EC thickness changes posttreatment as the outcome variable and *APOE4*, DHA treatment, and lipid changes as the explanatory variables, with the addition of sex and body mass index (BMI) as covariates in some models. If a variable needed to be standardized, the variable was divided by its SD. *P*-values below 0.05 (two-sided) were considered statistically significant. Multiple testing correction was not performed as the results were primarily exploratory given the relatively small sample size (n = 22). We, therefore, will not be able to draw generalizable conclusions from the results, but these data may inform hypotheses for future work. The analysis was done on a modified intention-to-treat basis (limited to trial completers with available follow-up outcome measures), and all subjects were included regardless of adherence. All statistical analyses were completed using R (http://www.R-project.org/).

## RESULTS

### Study sample description

Information regarding *APOE4* carrier status, gender, race, clinical dementia rating, age, BMI, and years of education from 22 participants were used in the analysis, and they were randomized into a DHA supplementation treatment or placebo groups. A summary of baseline characteristics is presented in Table 1. All participants showed no signs of clinical dementia. A statistically significant difference in median BMI was noted between the treatment group and the placebo group [means, CI: 26.52 (24.54, 29.26) and 32.77 (31.00, 35.06) kg/m^2^ respectively, *P* = 0.007]. The treatment group also had proportionally more females than males compared with the placebo group.

### Lipid subspecies changes in the DHA treatment and placebo arms

**Fig. 1:**
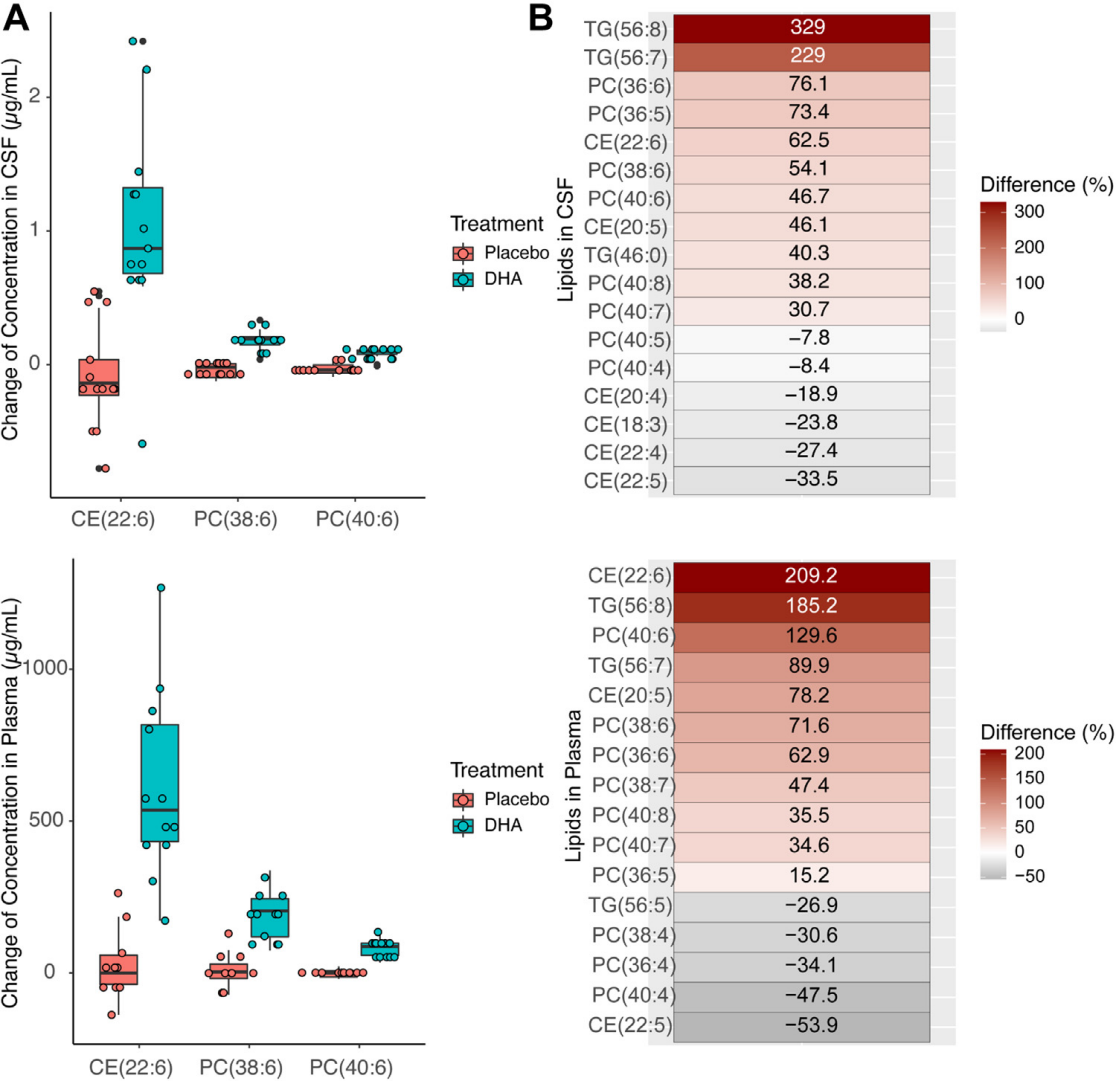
DHA-containing lipid level changes in CSF and plasma. A: Boxplots comparing mean changes in concentration levels of specific PC and CE lipid subspecies in CSF and plasma from baseline to follow-up in the DHA treatment group and placebo group. The specific lipid subspecies were selected from the top three 6-months average absolute concentration changes among all other lipids having *P*-values of <0.001 for the treatment effect in the linear model lipid(change) = lipid(baseline) + treatment. B: Ranking of the mean percentage changes of CSF (or plasma) lipid levels in the DHA-treated group minus mean percentage changes of CSF (or plasma) lipid levels in the placebo group. Mean percentage change is calculated as the mean of $\frac{lipid\left(change\right)}{lipid\left(baseline\right)}\times 100\%$ Specific lipid subspecies used in this ranking were selected based on having statistically significant changes from baseline to follow-up, adjusting for DHA treatment. The regression model used for selection was lipid(change) = lipid(baseline) + treatment.

At baseline, most DHA was found in CE in both CSF and plasma, followed by PC and with a smaller percentage in TG lipids. There was more DHA within TGs in plasma than CSF (supplemental Fig. S2). Following DHA supplementation, there were 20 lipid subspecies that changed between baseline after treatment compared with the placebo group (*P* < 0.05, uncorrected). These differences mostly occurred in TGs, PCs, and CEs among other types of lipids in CSF following DHA supplementation. The changes in CSF lipids based on the regression coefficient estimates are provided in supplemental Fig. S3. A list of the 20 DHA- and ARA-containing lipids can be found in Table 2. The major DHA-containing lipids by concentrations are shown in Fig. 1A. Consistent with the above results in CSF, DHA-containing lipids in plasma such as CE(22:6), TG(56:8), TG(56:7), and PC(40:6) increased in the treatment arm. Meanwhile, ARA-containing lipids such as PC(40:4) and PC(36:4) decreased. Notably, CE(22:5), likely to contain DPA, decreased in the DHA group compared to the placebo group. The DHA- and ARA-containing lipid changes were also ranked from highest to lowest based on the differences between mean percentage changes in the DHA group and placebo group (Fig. 1B). For CSF, TG-containing DHA TG(56:8) and TG(56:7) showed the largest percent increase, followed by CE(22:6) and PC(38:6), PC(40:6), PC(40:7), and PC(36:6). In contrast, CE(20:4), CE(18:3), CE(22:5), and PC(40:4) had an overall decrease after treatment. These results reflect the competition of DHA and ARA among some of these lipid pools and are consistent with increasing the DHA content of CE and PC lipids in CSF and the resulting significant decrease in the ARA content of CE (Table 3). Such inverse relationships between DHA and ARA-containing lipids were also confirmed by the grouped lipids analysis results, where increases in concentrations in the same DHA-containing CEs and PCs were complemented by decreases in concentrations in ARA-containing CEs and PCs in plasma (Table 3). These results were consistent with the previously mentioned inverse relationship between DHA and ARA which are likely to compete for the sn-2 position within the PL, and likely include DPA (17). The changes in all the lipids measured in CSF and plasma are shown by heatmaps in supplemental Figs. S4–S6.

**Table 2:** A list of DHA, ARA and EPA-containing lipids based on data collected using tandem mass spectrometry

DHA-containing lipids	CE 22:6, TG 52:6, TG 54:6, TG 54:7, TG 56:7, TG 56:8, TG 58:7, PC 38:6, PC 38:7, PC 40:6, PC 40:7, PC 40:8
ARA-containing lipids	CE 20:4, TG 56:5, PC 34:4, PC 36:4, PC 38:4, PC 38:5
EPA-containing lipids	CE 20:5, PC 36:5

ARA, n-6 arachidonic acid; CE, cholesteryl ester; DHA, n-3 docosahexaenoic acid; EPA, eicosapentaenoic acid; PC, phosphatidylcholine; TG, triglyceride.

The classification was first based on the lipid structure, if it contained 22:6, it was DHA-containing lipid, if it contained 20:4, it was ARA-containing lipid, and if it contained 20:5, it was EPA-containing lipid.

**Table 3:** DHA/ARA/EPA grouped lipids analysis as a function of treatment and *APOE* genotype

Sample	Group	CE	PC	TG
Treatment effect: Lipid (change) = treatment group + lipid (baseline)				
CSF	DHA	**1.125 (0.0001)**	**0.349 (<0.0001)**	0.016 (0.552)
	ARA	**−2.325 (0.014)**	−0.201 (0.127)	0.003 (0.492)
	EPA	**0.751 (0.008)**	**0.026 (0.0007)**	
Plasma	DHA	**548.008 (<0.0001)**	**285.783 (<0.0001)**	**51.759 (0.018)**
	ARA	−63.956 (0.663)	**−142.864 (0.007)**	**−1.679 (0.027)**
	EPA	**247.577 (0.003)**	**52.063 (0.024)**	
*APOE4* effect: Lipid (change) = *APOE4* + lipid (baseline)				
CSF	DHA	0.118 (0.729)	−0.042 (0.618)	**−0.054 (0.014)**
	ARA	0.880 (0.380)	−0.127 (0.276)	−0.007 (0.068)
	EPA	−0.327 (0.279)	−0.012 (0.159)	
Plasma	DHA	99.171 (0.550)	−25.300 (0.746)	−16.994 (0.500)
	ARA	252.993 (0.091)	−15.300 (0.778)	0.065 (0.947)
	EPA	−8.203 (0.928)	−29.095 (0.186)	
Interaction effect: Lipid (change) = treatment + *APOE4* + treatment group ∗*APOE4* + lipid (baseline)				
CSF	DHA	0.219 (0.671)	−0.005 (0.953)	−0.024 (0.572)
	ARA	0.520 (0.772)	0.124 (0.639)	−0.006 (0.396)
	EPA	0.711 (0.165)	−0.004 (0.707)	
Plasma	DHA	50.201 (0.817)	8.938 (0.928)	**−87.712 (0.029)**
	ARA	0.595 (0.998)	38.718 (0.680)	−0.488 (0.752)
	EPA	97.889 (0.514)	−21.546 (0.575)	

*APOE4*, apolipoprotein ε allele 4; ARA, n-6 arachidonic acid; CE, cholesteryl ester; DHA, n-3 docosahexaenoic acid; EPA, eicosapentaenoic acid; PC, phosphatidylcholine; TG, triglyceride; CSF, cerebrospinal fluid.

Grouped lipids analysis based on whether the lipid contains DHA, ARA, or EPA (See Table 2). After grouping into DHA, ARA, or EPA-containing lipids, the total baseline or change in concentrations in each group is summed within each participant. These sums were then fitted into one of the three regression models to test for possible treatment effect, *APOE4* effect, or interaction effect. The numbers in each cell represent the beta coefficient of the variable of interest (treatment, *APOE4*, or interaction of treatment and *APOE4*) and its *P*-value. *P*-values <0.05, uncorrected are in bold.

### Difference of lipid subspecies 6-months trial changes in plasma between DHA treatment and placebo groups

**Fig. 2:**
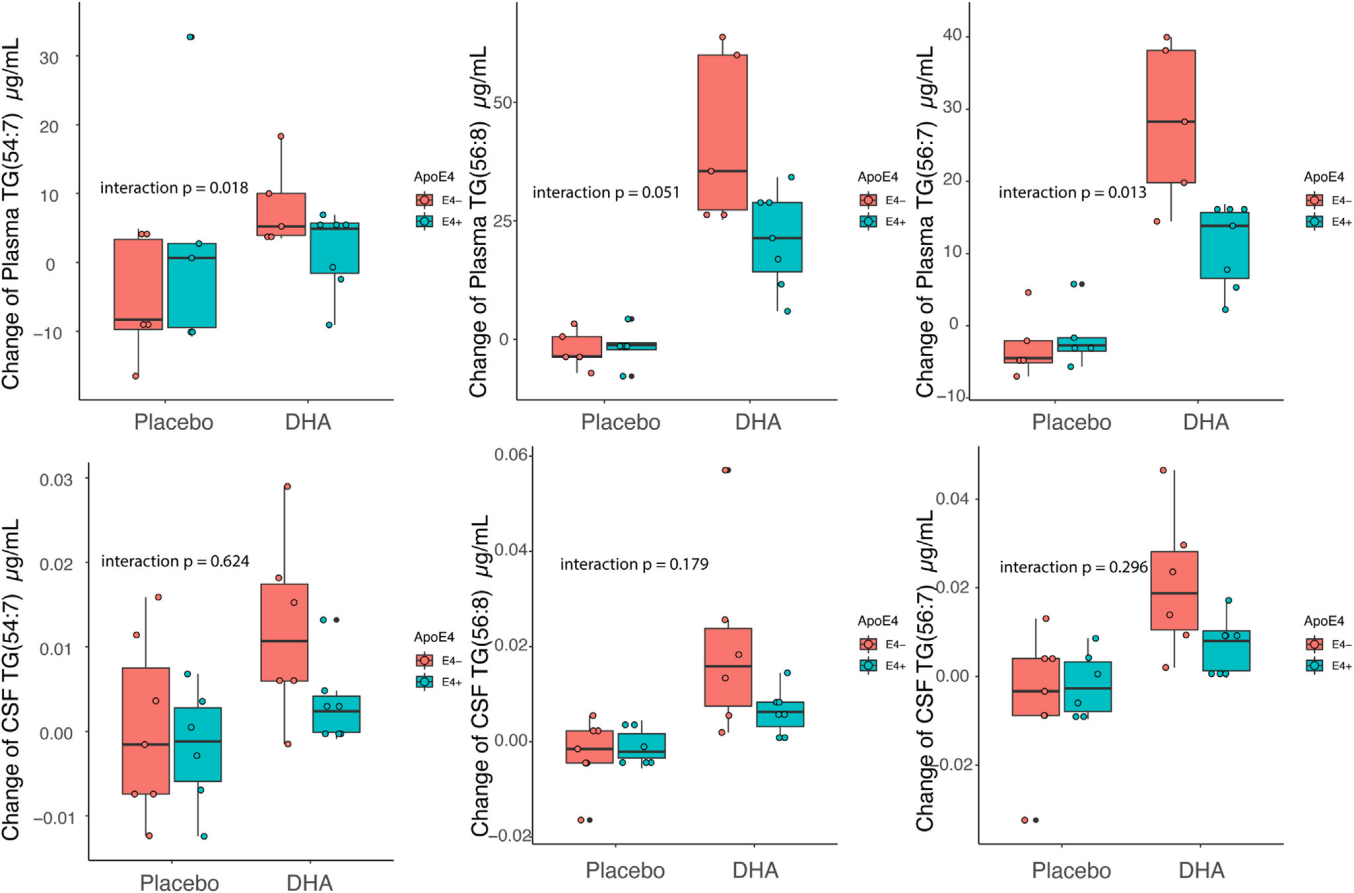
Changes in DHA-containing TG subspecies in plasma and CSF. *APOE4* carriers showed less increase in DHA containing TG subspecies levels in plasma and CSF following DHA treatment. The interaction model used was lipid(change) = treatment + *APOE4* + treatment∗*APOE4* + lipid(baseline). The *P*-values for the interaction term are indicated in each figure.

Consistent with the above results in CSF, DHA-containing lipids such as CE(22:6), TG(56:8), TG(56:7), and PC(40:6) also showed a greater increase between the DHA and placebo groups in plasma. Meanwhile, ARA-containing lipids such as PC(40:4) and PC(36:4) had overall negative changes between the DHA group and placebo group (Fig. 2). Such inverse relationships between DHA and ARA-containing lipids were also confirmed by the grouped lipids analysis results, where increases in concentrations in the same DHA-containing CEs and PCs were complemented by decreases in concentrations in ARA-containing CEs and PCs in plasma (Table 3). The results from CSF and plasma observed between the DHA treatment group are consistent as shown by heatmaps of lipid species. As DHA-containing lipids increased in CSF, they also increased in plasma, and as ARA-containing lipids decreased in CSF, they also decreased in plasma.

### The effect of *APOE4* on DHA-containing lipids in plasma and CSF

**Fig. 3:**
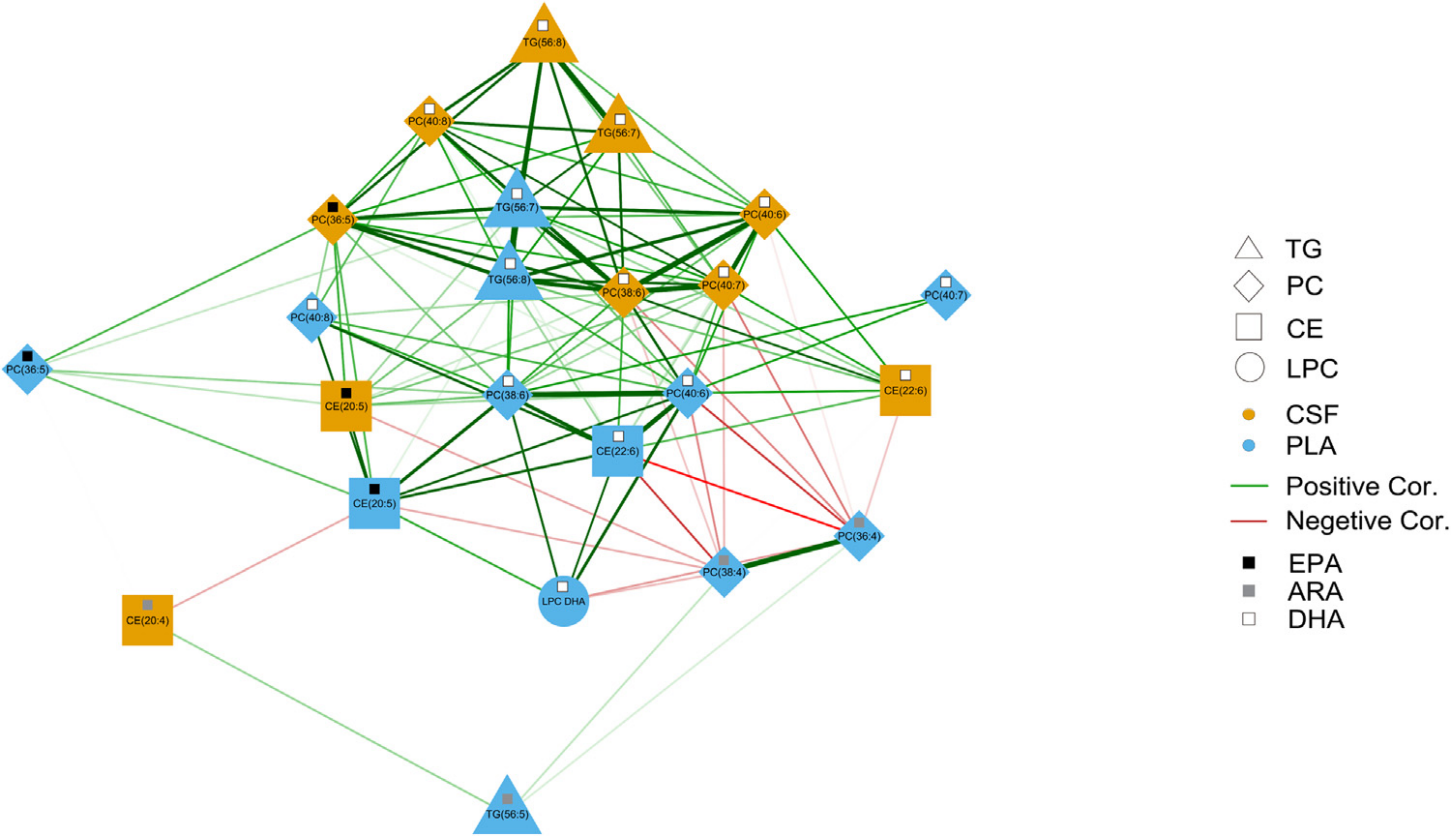
Correlation network of CSF and plasma lipids. Correlation network of the change (defined as 6-months follow-up minus baseline concentration) in DHA, ARA, and EPA-containing lipids in CSF and plasma. Green lines represent positive correlations, while red lines represent negative correlations. The thickness of the lines indicates the strength of the correlation. The node shapes represent species of lipids (square: CE; diamond: PC; triangle: TG; circle: LPC). Yellow nodes indicate lipids in CSF while blue nodes indicate lipids in plasma. The lipids selected for this network were significantly affected by DHA treatment according to having significant *P*-values of treatment beta coefficient in the model change = baseline + treatment. The pairwise correlations selected for this network were statistically significant. The pairs with absolute values of Pearson’s correlation coefficients less than 0.5 are hidden from view. DHA-containing lipids are marked with white square symbols, and ARA-containing lipids are marked with black square symbols.

In plasma, there was an interaction between *APOE4* carrier status and the treatment arm on DHA-containing TGs which responded to the 6-months DHA treatment but not on the other lipid species. Regression models with interaction terms between *APOE4* and treatment showed *APOE4* carriers on treatment had a lower increase in TG(54:7), TG(56:8), and TG(56:7) compared to *APOE4* noncarriers (Fig. 3A). The effect on CSF was similar. However, the interaction term was not significant at a *P* < 0.05, likely due to the greater variability with the lower CSF TG levels. Similar results were found in grouped lipids analysis (Table 3). DHA-containing TGs in plasma had reduced positive changes in *APOE4* carriers compared to *APOE4* noncarriers following DHA treatment (interaction beta = −26.67, *P* = 0.029). These results suggest that *APOE*4 may affect TG PUFA metabolism impacting brain DHA delivery after supplementation.

### Network analysis of lipid subspecies and DHA and ARA in CSF and plasma

**Fig. 4:**
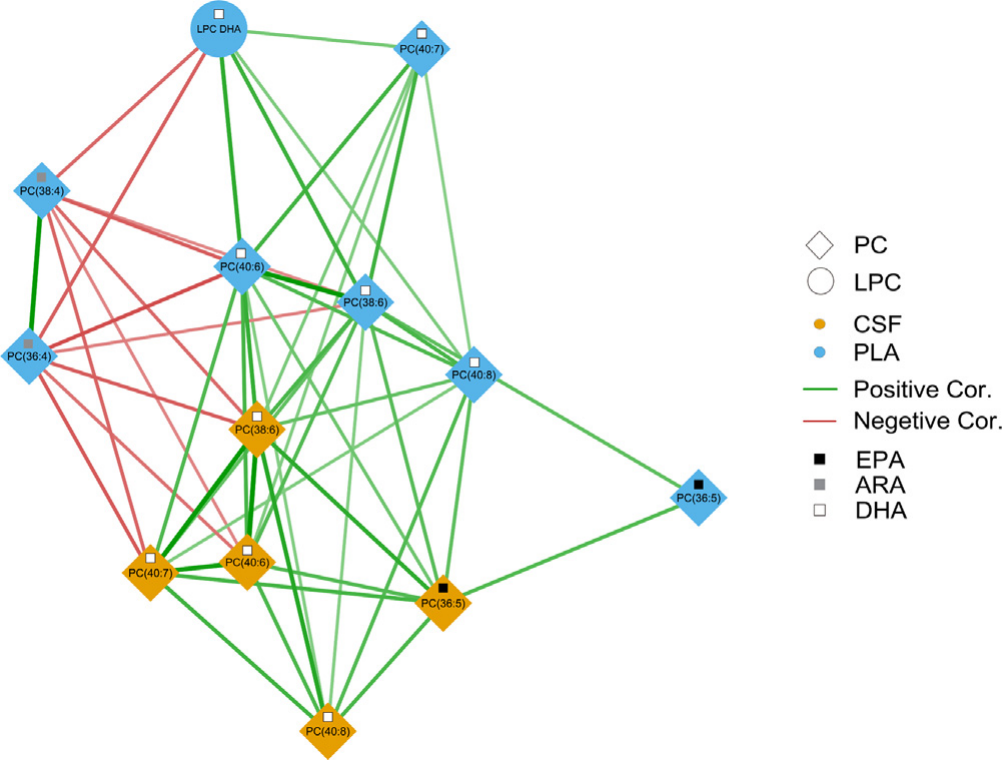
Correlation network of LPC and PC lipids. Correlation network of the change (defined as 6-months follow-up minus baseline concentration) in DHA, EPA, and ARA contained in LPC and PC lipids in CSF and plasma. Green lines represent positive correlations while red lines represent negative correlations. The thickness of the lines indicates the strength of the correlation. Yellow nodes indicate lipids in CSF, while blue nodes indicate lipids in plasma. The lipids selected for this network were significantly affected by DHA treatment according to having significant *P* values of treatment beta coefficient in the model change = baseline + treatment. The pairwise correlations selected for this network were statistically significant. DHA-containing lipids are marked with white square symbols. ARA-containing lipids are marked with black square symbols.

Network analysis of lipid changes (6-months follow-up measurements minus baseline measurements) in plasma and CSF showed robust clustering of DHA-containing lipids within each other and negatively with ARA-containing lipids. The changes observed in CE, PC, and TG DHA-containing lipids such as TG(56:7), PC(38:6), and CE(22:6) were strongly and positively correlated with each other within CSF and plasma (Fig. 3), implying efficient DHA flux within the different pools of circulating lipids, many of which (PCs, LPCs, and CEs) are enriched on HDL particles following consumption of TG-based DHA supplement. Negative correlations among subspecies were observed in changes in ARA-containing lipids such as PC(36:4), PC(40:4), and PC(38:4). As expected, LPC-DHA was positively correlated with DHA-containing PC lipids in CSF and plasma (Fig. 4) such as PC(40:6) and PC(38:6). The DHA-containing TG lipids within very long chain fatty acids were strongly correlated between CSF and plasma with R = 0.83 for TG(56:8) (supplemental Fig. S6) raising the possibility of direct transfer across the blood-CSF barrier.

### CSF HDL changes following treatment

**Fig. 5:**
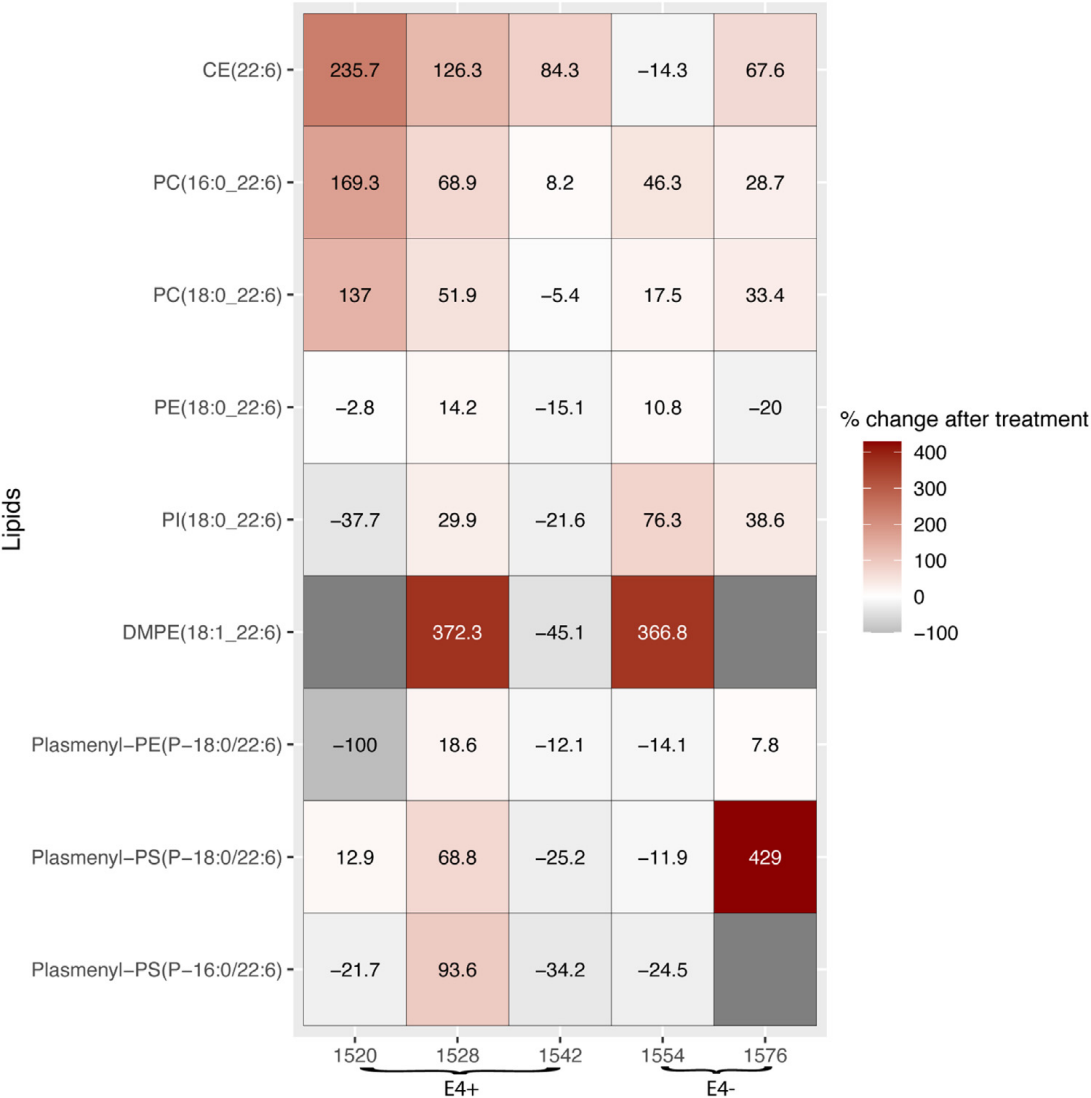
HDL lipid changes after treatment. HDL particle concentrations were measured in five subjects and the percentage change in DHA-containing lipids after treatment was compared between *APOE4* carriers and noncarriers.

Lipoprotein particles in CSF were isolated by sequential differential centrifugation, and lipids were measured in five subjects before and after treatment and randomly sampled from *APOE4* carriers and noncarriers in the DHA arm. Lipids in the density of HDL were detected. The main lipids that increased in those subjects were CE(22:6), PC(38:6), and PC(40:6) in CSF, confirming that particles in the density of HDL are carriers of DHA in the brain following supplementation (Fig. 5). There was an overall decrease in ARA-containing lipid levels after treatment (supplemental Fig. S7).

### EC thickness changes following DHA supplementation and effect of *APOE4*

**Fig. 6:**
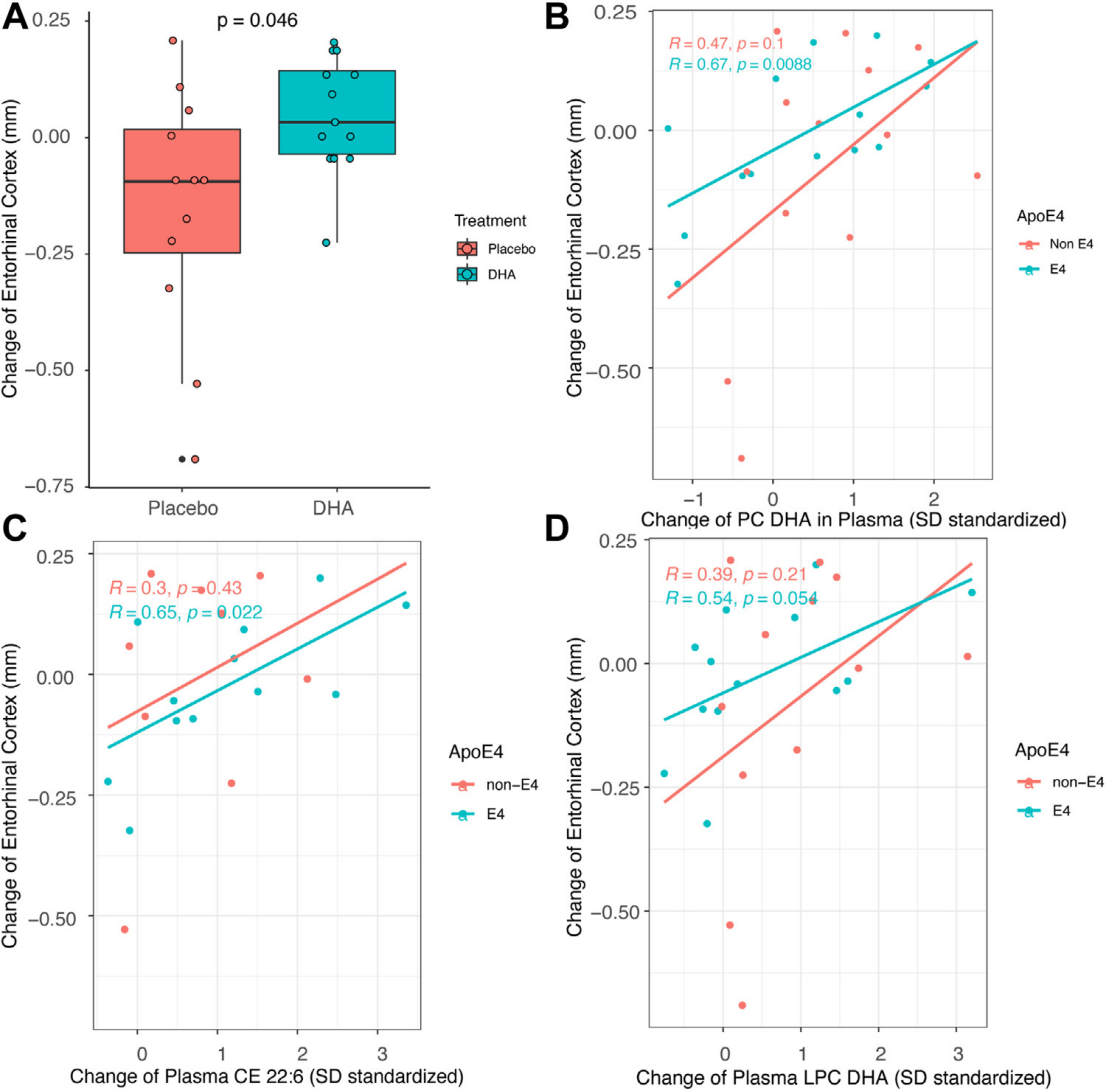
The effects of treatment and DHA-containing lipids on the entorhinal cortex. A: DHA treatment, adjusted for *APOE4*, was associated with a significant increase in the entorhinal cortex area thickness compared with placebo. Regression model: ERC change = treatment + *APOE4* [mean group difference in ERC change (95% CI) 0.161 (0.003, 0.319), *P* = 0.046]. The same statistically significant results were observed for the treatment effect when additional sex and BMI were added. Regression model: ERC change = treatment + *APOE4* + BMI + sex (mean group difference in ERC change (95% CI) 0.164 (−0.05, 0.38), *P* = 0.03). B: Regression of SD-standardized plasma PC DHA (total PC DHA) concentration and entorhinal cortex thickness change after adjusting for *APOE4*. C: The entorhinal cortex thickness was associated with a change of plasma CE (22:6), which is a DHA-containing lipid, after adjusting for *APOE4*. D: Change in entorhinal cortex thickness was associated with the change of plasma LPC DHA. SD standardization was done by dividing the variable by its standard deviation.

DHA treatment was associated with greater EC thickness compared with placebo (*P* = 0.046, adjusted to *APOE4*, Fig. 6A). Similar results were observed for the treatment effect when additional covariates, BMI, and sex were added to the model. Among all the DHA-containing lipids, plasma PC-DHA and CE-DHA had stronger correlations than plasma LPC-DHA with the EC thickness change, independent of *APOE4*. As DHA within PC and CE levels in plasma and CSF increased, the change in EC thickness increased in both noncarriers and carriers of *APOE4* (Fig. 6B–D).

**Fig. 7:**
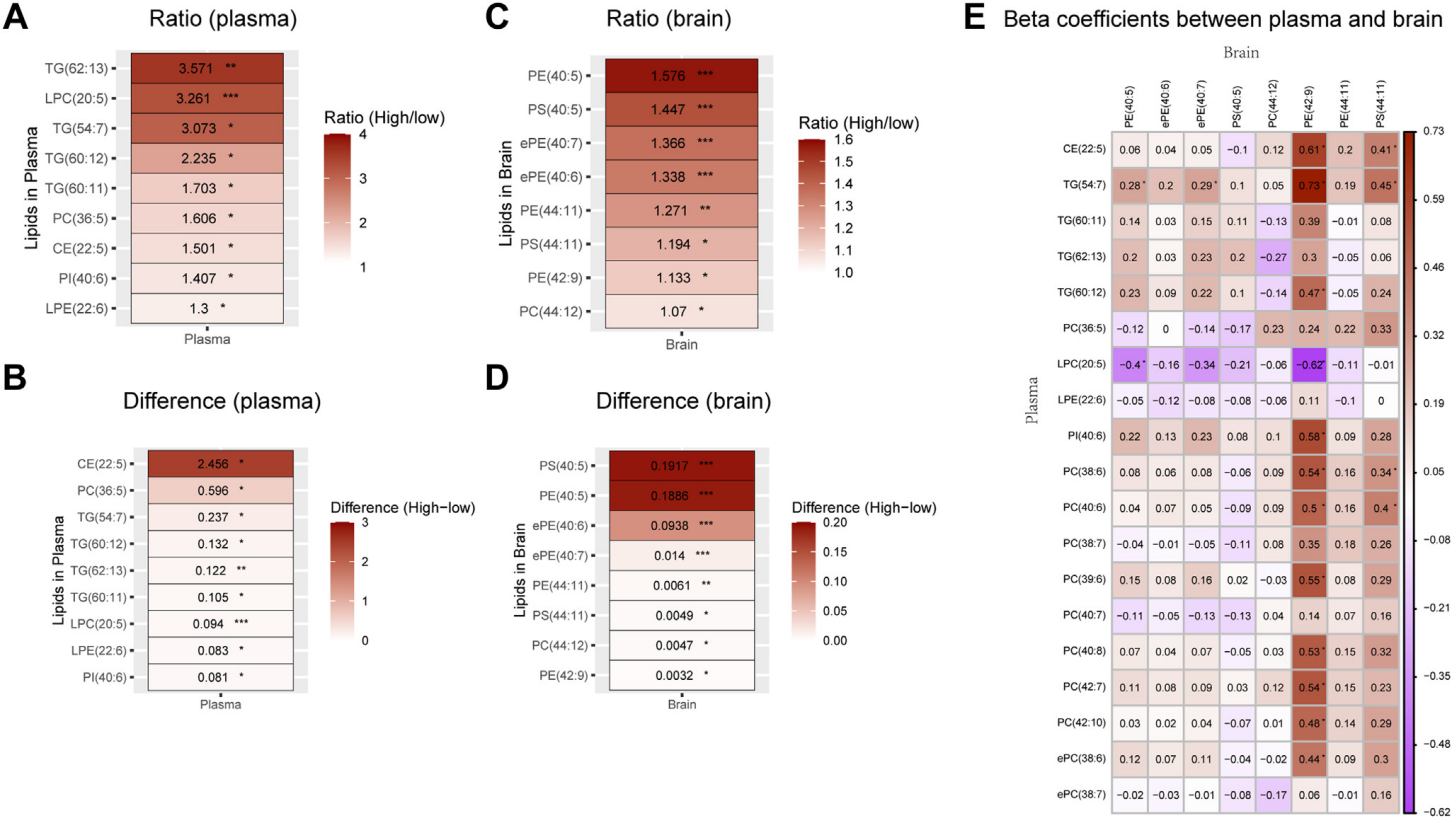
Lipid level changes in CSF and plasma from mice on low versus high DHA diet. A: The ratio ordering of plasma lipids with significant differences between high DHA groups and low DHA groups. B: The difference ordering of plasma lipids with significant differences between high DHA groups and low DHA groups. C: The ratio ordering of brain lipids with significant differences between high DHA groups and low DHA groups. D: The difference ordering of brain lipids with significant differences between high DHA groups and low DHA groups. The ratio is defined as the mean(high)/mean(low), difference = mean(high) -mean(low). E: The standardized beta coefficients obtained from the linear regression model between brain lipids and plasma lipids, with groups as the covariate. (∗*P* <0.05, ∗∗*P* < 0.001, ∗∗∗*P* < 0.0001).

*A high DHA diet illustrates the exchange of PUFAs between plasma and brain lipids:* A 4-months high DHA diet remodeled plasma and brain PUFA-containing lipids in wild-type C57B6/J mice. The mouse brain lipids responding to the higher DHA diet were predominantly consisting of phosphatidylethanolamine PE (40:5) and PE (42:9) (Fig. 7A, B). In plasma, the DPA-containing CE(22:5) yielded the greatest difference, while the DHA-containing TG(62:13) resulted in the greatest ratio change between high and low DHA diets (Fig. 7C, D). Modeling the brain lipids as a function of plasma lipids adjusted for treatment groups revealed a striking association, assessed using the standardized beta coefficients, between several classes of plasma lipids containing DHA, EPA, and DPA with PUFAs contained in brain phosphotidylethanolamine (PE) and phosphatidylserine lipids (Fig. 7E).

## DISCUSSION

In the DHA Brain Delivery Pilot trial, we demonstrated a 28% increase in CSF DHA levels after dietary supplementation with 2,152 g per day of algal TG-based DHA supplements over a period of 6 months (2). The goal of this study was to investigate the changes in lipid species containing DHA and examine their relationship to the DHA-rich EC area of the brain. After 6 months of treatment, DHA contained within PC, CE, and TG lipid species all increased. The correlation of PC and CE DHA with the EC thickness implicates a role for HDL particles as a source or biomarker of brain DHA. The enrichment of DHA within HDL lipids was directly confirmed by isolating particles from CSF by centrifugation at the expected density of HDL before and after supplementation.

We observed a strong correlation of DHA-containing lipids in both the CSF and plasma suggesting either an exchange of HDLs at the blood-CSF barrier or shared mechanisms forming HDL particles in both compartments (12). Furthermore, the increase in DHA was found to be consistent with the replaced ARA and DPA within the same lipid pools, confirming the competition of omega-3s and omega-6s at the sn-2 position of PLs (6). The strong correlation between the changes of DHA containing TG’s in the CSF and plasma merits discussion. There have been limited studies on TGs in CSF, primarily due to their low abundance (17). It is possible that TGs in CSF exist in larger particles containing ApoE, but we were not able to detect them following centrifugation at the density of very low density lipoproteins (VLDL). It is plausible that DHA-containing TGs could be entering the brain either from chylomicrons formed after absorption or VLDLs excreted from the liver, or both. In a study that investigated DHA uptake using radioactive tracers, the liver selectively captured labeled DHA uptake and secreted it into TGs (18). There is a strong possibility that very long chain fatty acid within TGs may enter the brain directly, escaping liver metabolism (17). The transport of DHA on plasma TGs is of significance to AD pathology. In an Alzheimer’s Disease Neuroimaging Initiative study, levels of PUFAs on TGs correlated with both the EC thickness and CSF Aβ1-42 levels, with a stronger correlation observed in E4 carriers than noncarriers (9).

The DHA Brain Delivery Pilot trial tested the effect of *APOE4* on omega-3 brain delivery, revealing differences in total DHA and EPA CSF levels by genotype. Here, *APOE4* had the strongest effects on the DHA-containing TG lipid pools, typically found in larger particles (VLDL and chylomicrons). The increase in DHA within TGs was suppressed in *APOE4* carriers compared to noncarriers in CSF and plasma. One mechanism for this observation is the increased oxidation of DHA-containing TG particles in *APOE4* cells, previously shown using 13-C DHA tracer studies in humans (19). Alternately, *APOE4*-containing endogenous particles may alter the transport or metabolism DHA containing lipids compared to *APOE2* and *APOE3*. One study investigating astrocyte uptake of TGs between *APOE4* carriers and noncarriers demonstrated differential metabolism of fatty acids based on genotype (20). In another study involving supplementation of a vitamin A-containing fatty diet and a vitamin A-fat loading test, *APOE4* carriers had a faster clearance of lipids than *APOE2* and *APOE3* carriers (21). Together, these findings suggest that *APOE4* causes either deficient accretion of PUFAs or increased clearance, and this may diminish the effects of supplemental omega-3 on the brain.

There is strong interest in identifying the form of supplemental DHA that best enriches the brain (22), with mouse studies suggesting that LPC DHA formulation is superior to TG-based DHA (23). Although the participants in this study consumed TG-based algal DHA, it is known that TGs are hydrolyzed in the intestine and absorbed in free fatty acid form and reconverted into TG and PC lipid pools either following absorption or after secretion from the liver. We found that the changes in DHA transported on TG, CE, and PC lipids were highly correlated and furthermore that changes in PCs and LPC DHA levels were strongly correlated, reflecting a rapid flux between the different PL compartments. It is surprising that the changes of PC DHA in plasma showed a stronger correlation with the change in EC thickness than CSF PC DHA, as CSF is a better marker of brain lipids than plasma. However, this weaker association could have been a function of the small sample size together with the much lower concentrations in CSF PC DHA than in plasma. In addition, CSF PE DHA may be a better surrogate of brain DHA than CSF PC DHA, as shown in the mouse study (Fig. 7). Unfortunately, we were not able to accurately measure DHA contained in CSF PEs in the human study. Overall, DHA levels were correlated within different lipid species and across the plasma and CSF pools inferring a dynamic exchange of PUFAs into different PC and LPC pools. Among the brain compartments, the EC appears to be sensitive to a DHA-enriched diet and a previous animal study confirmed the importance of dietary DHA on preventing EC dysfunction (10). In plasma, we did not find that LPC DHA was a superior marker of the change in EC thickness compared to PC or CE DHA. However, this study was not designed to directly compared PC to LPC DHA formulations. Whether ingestion of a PL-based DHA results in greater enrichment of DHA within HDLs, LPCs, or the brain in humans remains to be determined.

Our study supports the hypothesis that DHA on HDL particles is a marker for DHA in the brain. DHA within HDL may reflect the DHA content of lipids in plasma membranes. HDLs share similarities to the plasma membrane lipidome composition (i.e., enriched in cholesterol and sphingomyelins) within lipid rafts, suggesting that newly formed HDLs may be derived from lipid rafts of plasma membranes (24). In the mouse study, we observed that higher plasma levels of PUFAs (DHA, EPA, and DPA) across all lipid classes (TG, PE, PC, and phosphatidylinositol) were correlated with higher levels of PUFA lipid species in the brain, most notably DPA-containing lipids (Fig. 7E). The mouse diet was formulated from DHA-S supplements (DSM), and unlike the human DHA diet, the mouse diet contained 19% n-6 DPA. The n-6 DPA has anti-inflammatory effects and was shown to have neuroprotective effects in *APOE4* AD mouse models (25). The MIDAS trial provided positive effects of DHA-S on cognitive outcomes (26). The ongoing randomized clinical trial PreventE4 (NCT03613844) is testing whether *APOE4* influences CSF DHA level after DHA-S supplementation (n=184) as the primary outcome, with MRI imaging and cognitive biomarkers (n=368) as secondary and exploratory outcomes, respectively.

Our study has a few limitations. The small study sample size limited its ability to look at the effects of DHA lipids and cognition. Despite the BMI and sex imbalance between the treatment and placebo groups, the treatment effect on the EC thickness remained significant at *P* < 0.05. We could not measure TGs in CSF fractions collected after centrifugation because of their lower abundance. Our future studies will include a substantially larger sample volume to address these particles. The MS method was not able to accurately resolve phosphatidylethanolamine species. Finally, since we did not adjust for multiple comparisons, some correlations could have been observed by chance. We recognize that our findings are nominal in significance and exploratory in nature. A larger study (PreventE4, NCT03613844) is ongoing to validate and expand our findings. The DHA diets in the mouse study and the human study were not identical and this could limit the translation of the human and mouse study findings.

## Data availability

Deidentified or coded data will be shared upon request to the corresponding author after obtaining a data use agreement.

## Supplemental data

This article contains supplemental data.

## Conflict of interest

The authors declare that they have no conflicts of interest with the contents of this article.
