# Supplementary material for: Associations of ApoE4 status and DHA supplementation on plasma and CSF lipid profiles and entorhinal cortex thickness
Source: J Lipid Res. 2023 Mar 22;64(6):100354. doi: 10.1016/j.jlr.2023.100354 (PMC10230261; doi:10.1016/j.jlr.2023.100354)
Supplement: Supplemental data [file mmc1.pdf]

Supplementary File

Associations of ApoE4 status and DHA supplementation on plasma and CSF lipid profiles and entorhinal cortex thickness

Mikaila A Bantugan[1*], Haotian Xian[1*]; Victoria Solomon[1*], Mitchell Lee[1], Zhiheng Cai[1], Shaowei Wang[1], Marlon V. Duro[1], Alfred Fonteh[2], Cristiana Meuret[1], Meitong Li[1], Meredith N. Braskie[1], Laura Beth J. McIntire[3], Lucia Jurin[4], Sarah Oberlin[4], James Evans[4], Roderick Davis[4], Wendy J. Mack[5], Laila Abdullah**[4,6] and Hussein N. Yassine**[1]

[1] Departments of Medicine and Neurology, Keck School of Medicine, University of Southern California, Los Angeles, California, 90033

[2] Huntington Medical Research Institutes, Pasadena, California, 91105

[3] Department of Radiology, Brain Health Imaging Institute, Weill Cornell Medical College, 10065

[4] Roskamp Institute, Sarasota, Florida, 34243

[5] Department of Population and Public Health Sciences, Keck School of Medicine, University of Southern California, Los Angeles, California, 90033

[6] James A. Haley VA Hospital, Tampa, FL, 33612

* Equal contributions
** co-senior authors

Short title: DHA lipid species and Brain Delivery

Correspondence
Hussein Yassine, M.D.
Keck School of Medicine
University of Southern California
Los Angeles, CA, 90033
hyassine@usc.edu

Supplementary Tables 1

Supp Table 1 A. Fatty Acid composition of DHA and placebo in the human study		
**Fatty Acid**	DHA (%)	Placebo (%)
14:0	10.8	<0.1
16:0	7.8	11.2
18:0	0.2	2.7
18:1n-9	7.7	22.8
18:2n-6	<0.1	54.0
18:3n-3	<0.1	4.3
20:5n-3 (EPA)	<0.1	<0.1
22:0	<0.1	0.2
22:5n-3	0.9	<0.1
22:5n-6 (DPA)	<0.1	<0.1
22:6n-3 (DHA)	60.1	<0.1

Supp. Table 1: (B) Fatty Acid composition of DHA and placebo in the mouse study		
**Fatty Acid**	DHA (%)	Placebo (%)
14:0	5.4	<0.1
16:0	16.2	11.2
18:0	0.5	2.7
18:1n-9	4.5	22.8
18:2n-6	0.5	54.0
18:3n-3	<0.1	4.3
20:5n-3 EPA	1.7	<0.1
22:0	<0.1	0.2
22n:5n-3	0.6	<0.1
22:5n-6 DPA	19.4	<0.1
22:6n-3 DHA	43.7	<0.1

Supplementary Figure 1: Representative chromatograms of TG and PC containing lipids.

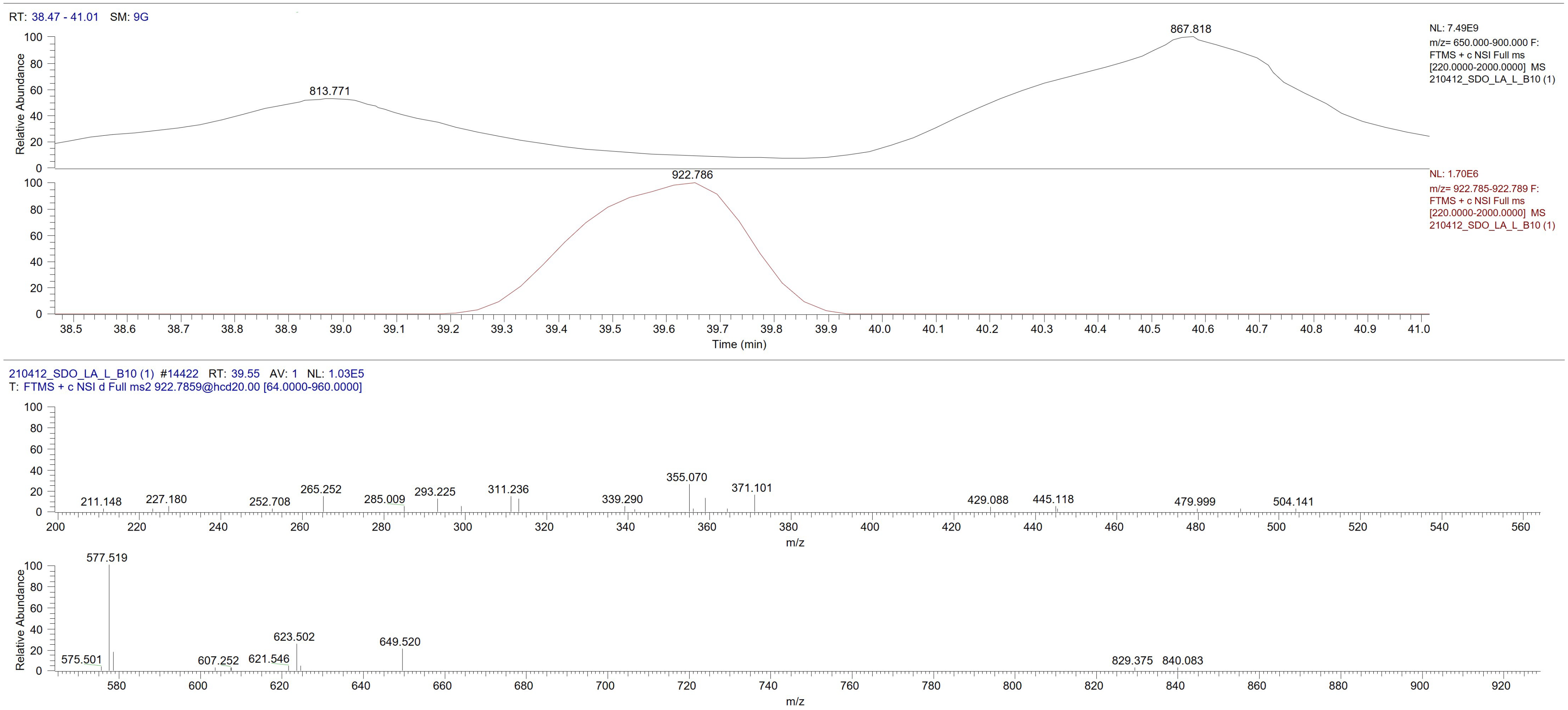

Supp Fig 1A: A positive ion mode M+NH4+ ammonium ion plots and corresponding MS/MS spectra of TG 16:0/18:1/22:6 in CSF. Fatty acid for DHA (FA22:6) is detected as neutral loss (NL) of FA22:6+NH3.

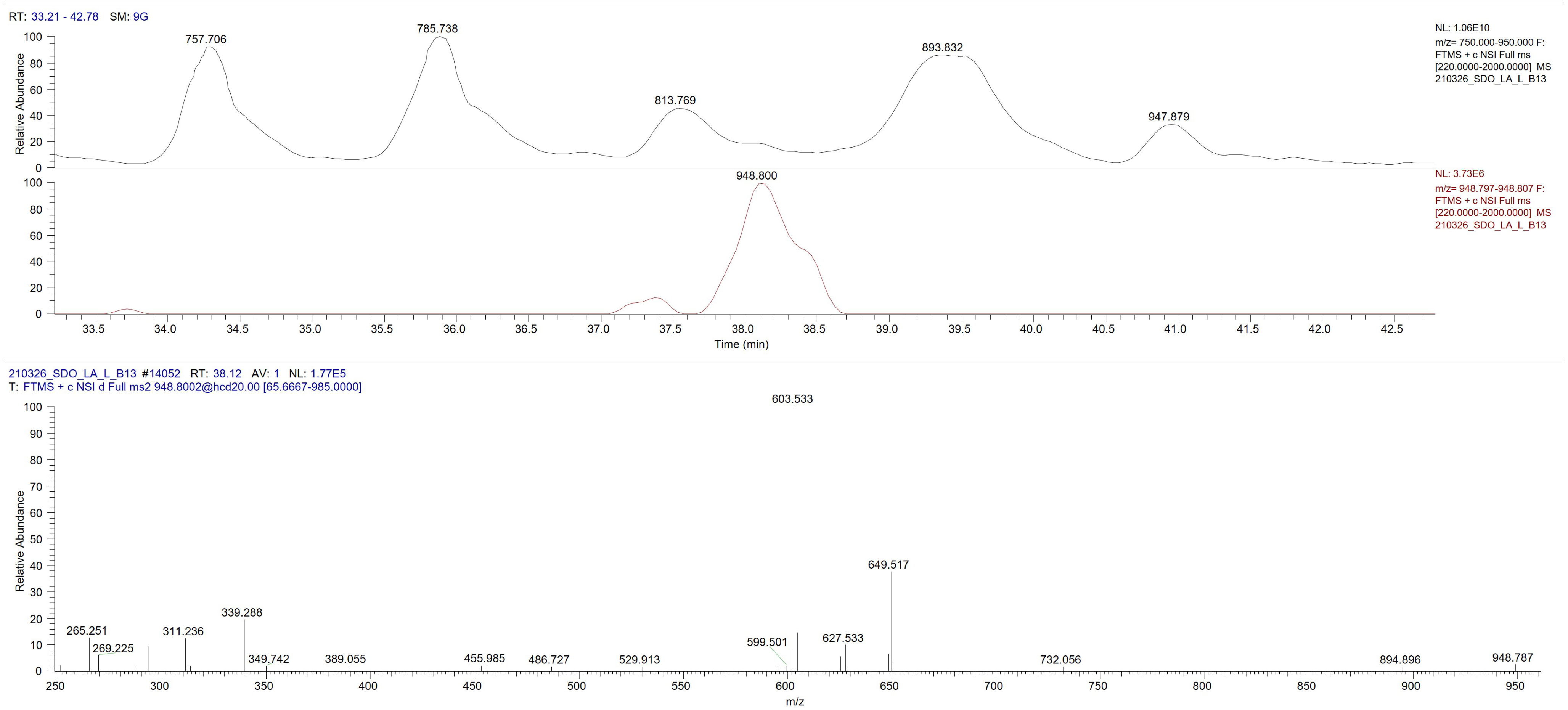

Supp Fig 1B: TG 18:1/22:6/18:1 in plasma is shown and fatty acid determination is made similar to panel A

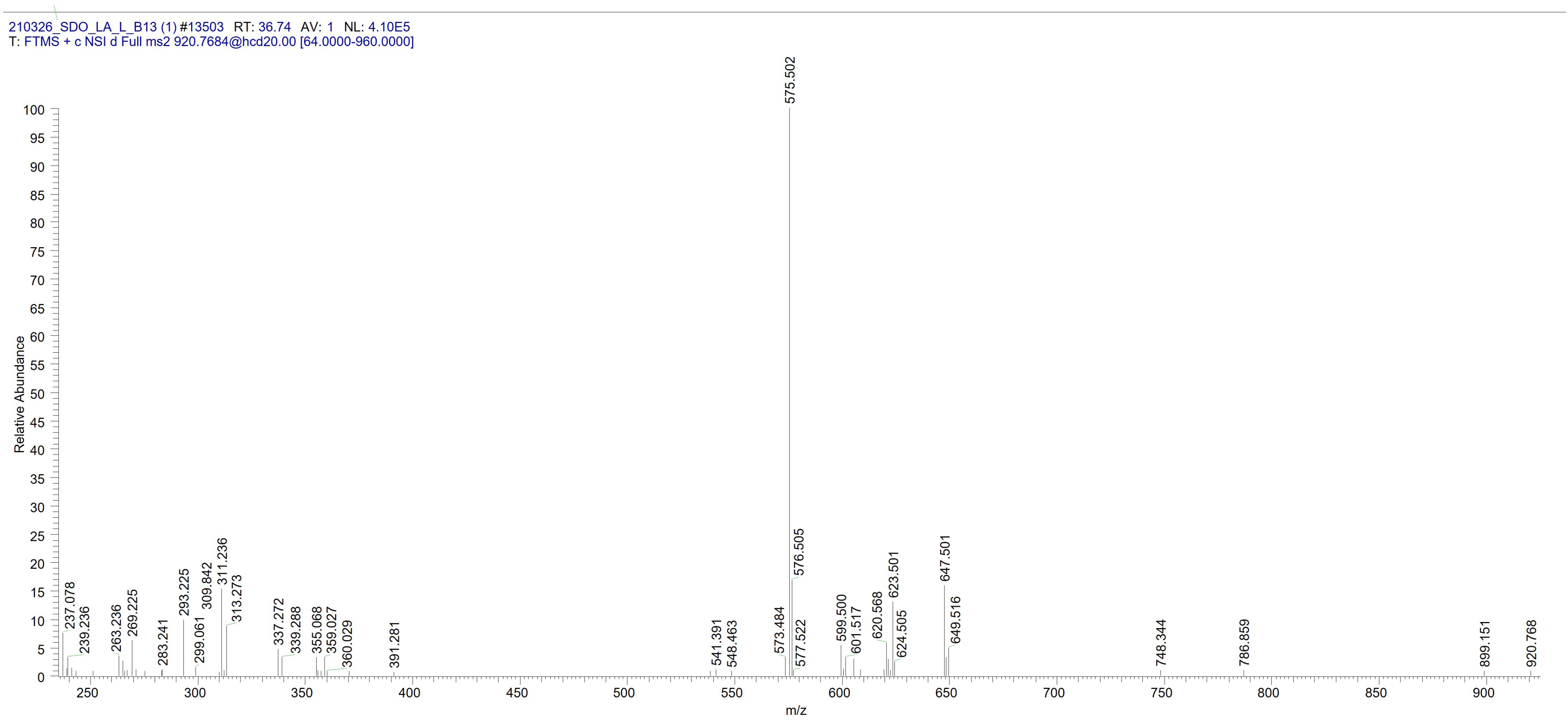

Supp Fig 1C: Major TG detected in HDL particles.

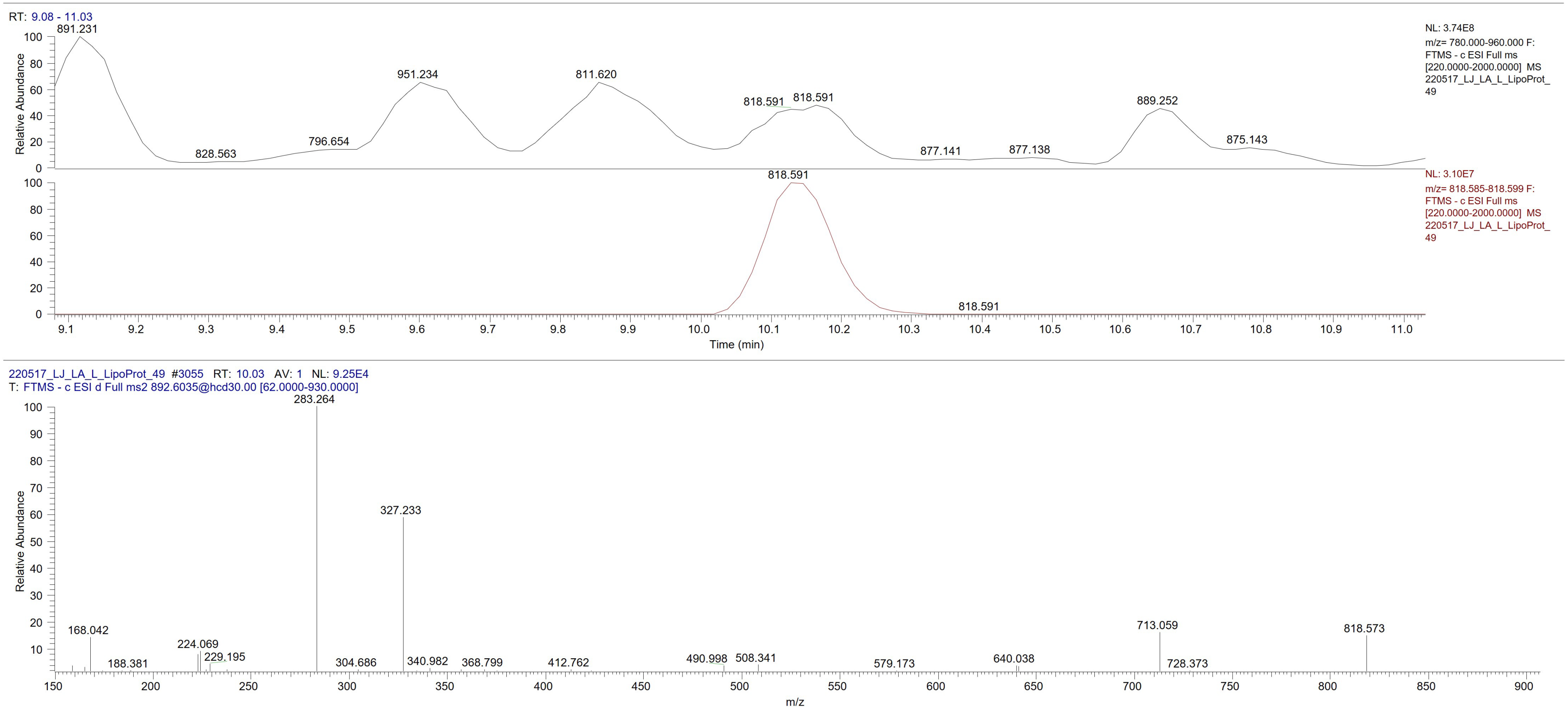

Supp Fig 1D: [M+acetate]- ion plots and corresponding MS/MS spectra for plasma PC species detected in the negative ion mode. Fatty acids are detected in as their RCOO- adducts in the negative ion mode.

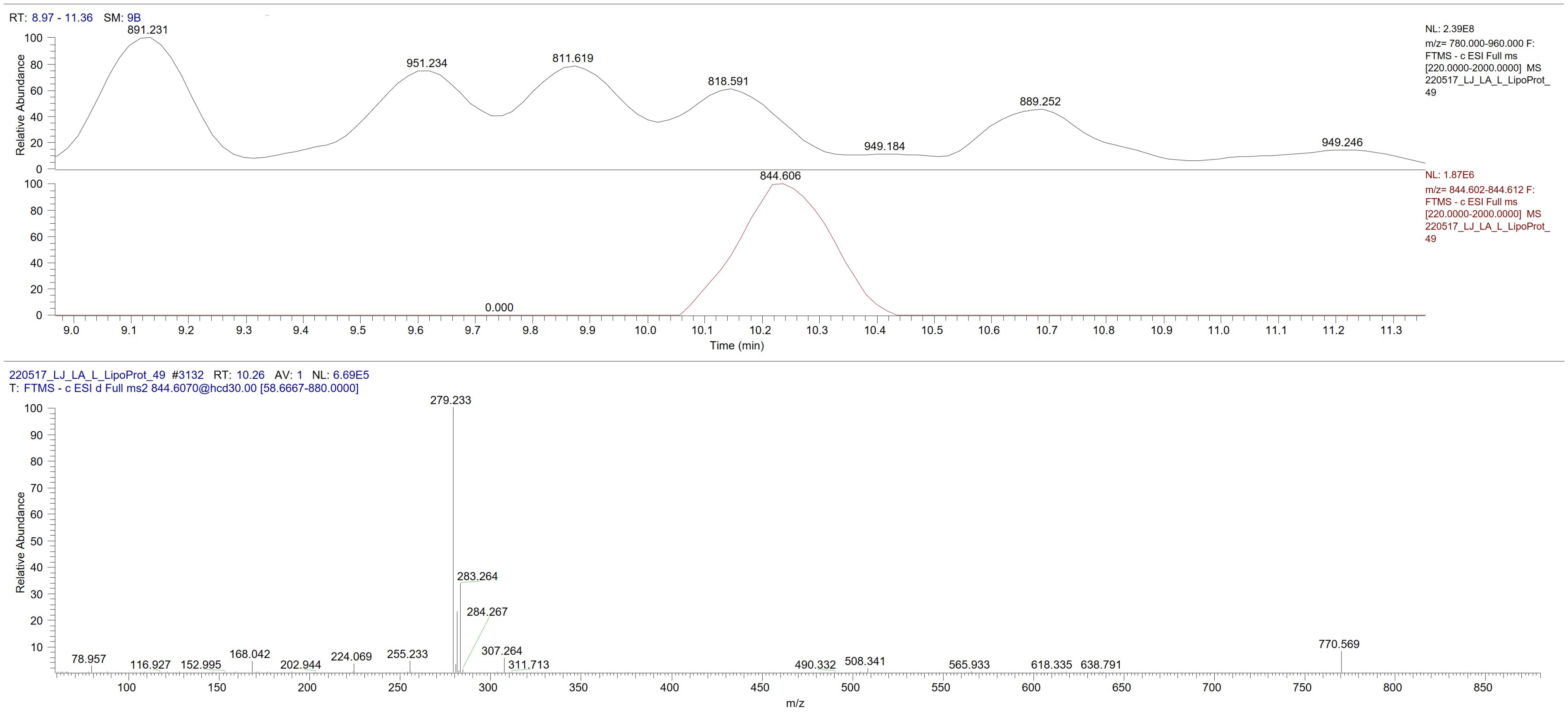

Supp Fig 1E: [M+acetate]- ion plots and corresponding MS/MS spectra for plasma PC species detected in the negative ion mode. Fatty acids are detected in as their RCOO- adducts in the negative ion mode.

Supplementary Figure 2: Baseline composition of DHA containing lipids in CSF and plasma

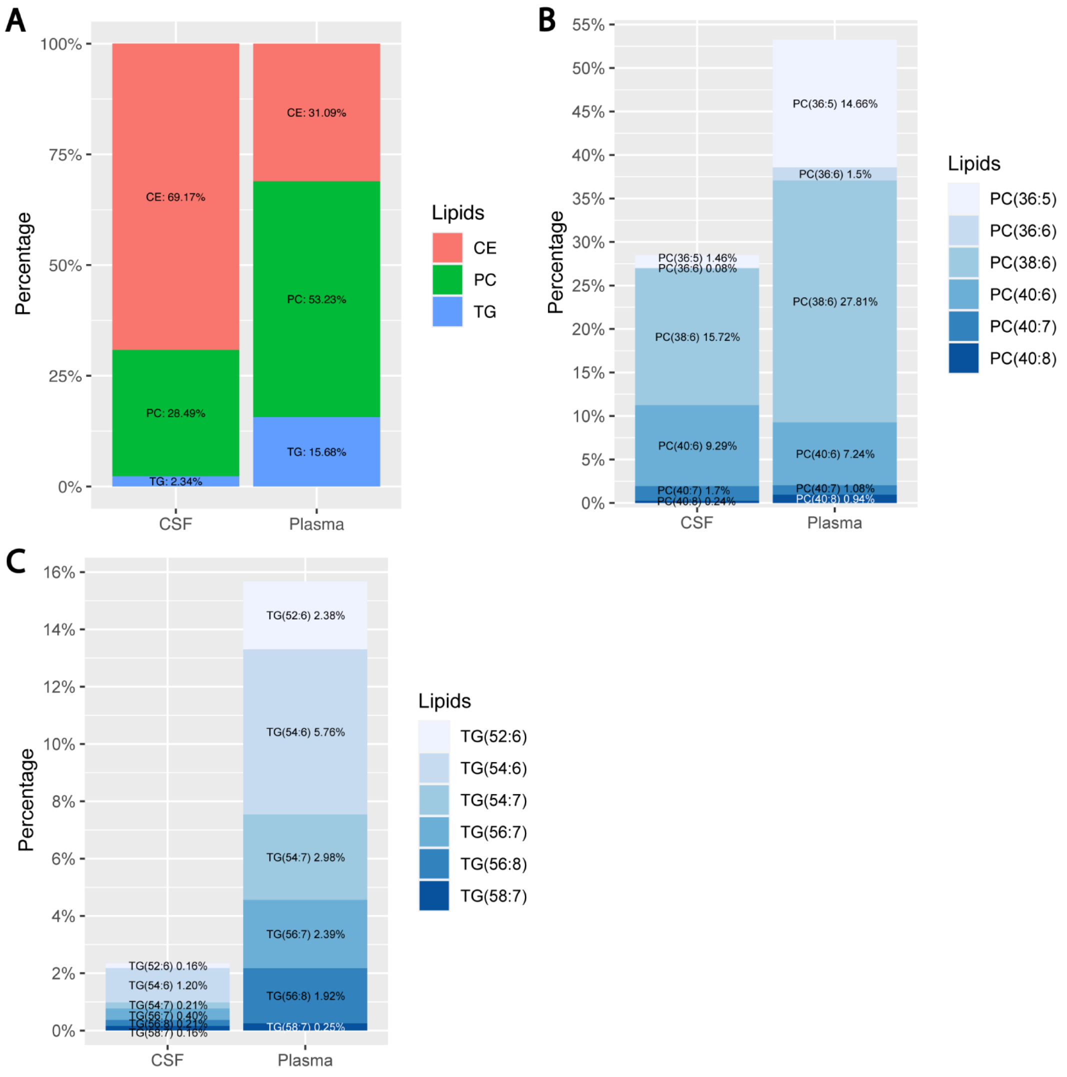

Supplementary Figure 2: Percentage compositions of baseline concentrations of DHA containing lipids of the types of CE, PC, and TG in CSF and plasma.

Supplementary Figure 3: Ranking of beta coefficients of the CSF lipid changes

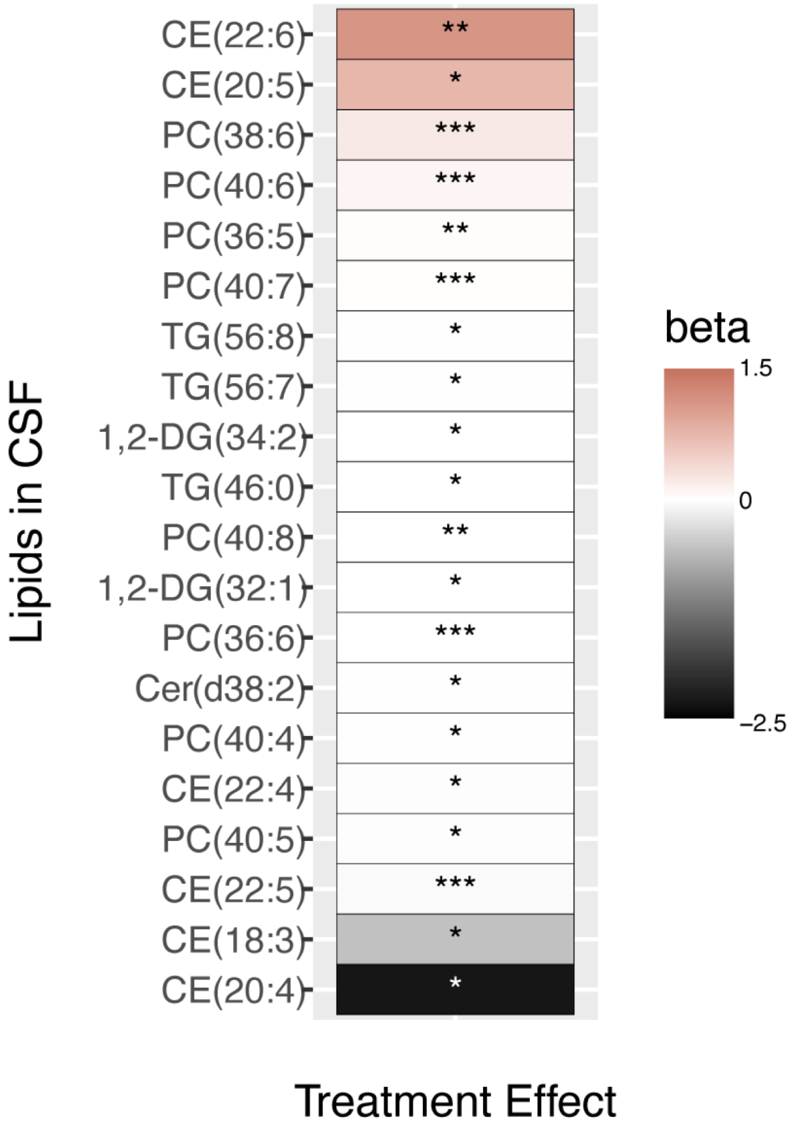

Supplementary Figure 3: Ranking of beta coefficients of the treatment variable from the linear model lipid(change)=lipid(baseline)+treatment, where change is calculated as a 6- month follow-up measurement minus baseline measurement. The asterisks represent the value of the p-value (* p <0.05, ** p <0.001, *** p < 0.0001).

Supplementary Figure 4: Correlation heatmaps of CSF and plasma of (A) CE lipids and (B) DG lipids change

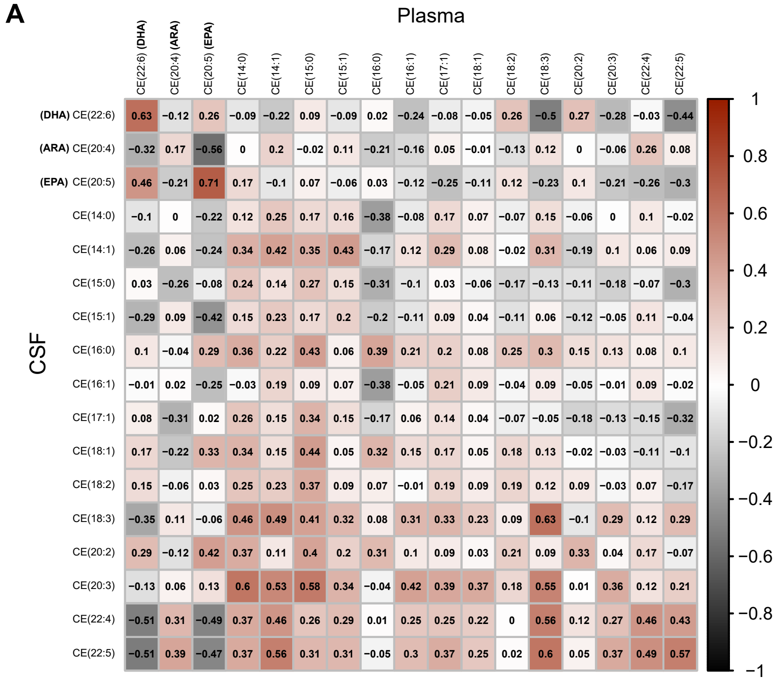

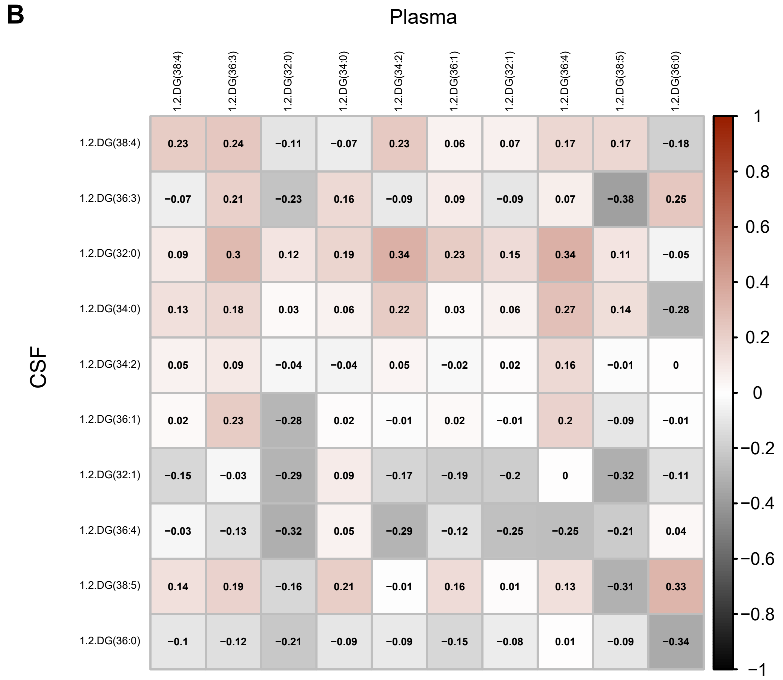

Supplementary Figure 4: Correlation heat maps between CSF and plasma lipid pools showing Pearson's correlation coefficient in each cell. The correlations were done using 6-month concentration changes in each subspecies. CE: cholesterol ester. DG: Diacyl-glycerol.

Supplementary Figure 5: Correlation heatmaps of CSF and plasma PC lipid change

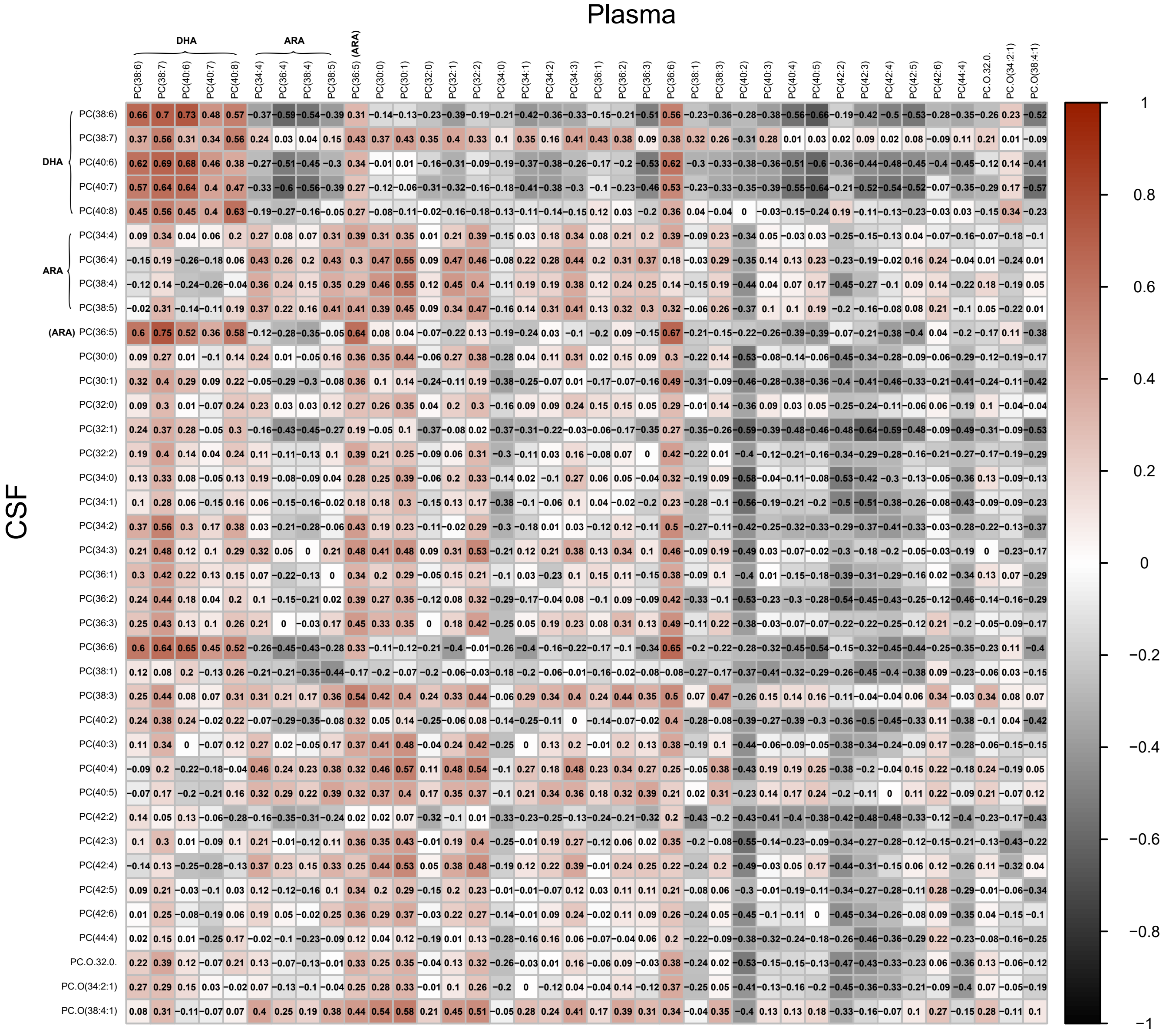

Supplementary Figure 5: Correlation heat maps between CSF and plasma lipid pools showing Pearson's correlation coefficient in each cell. Blue represents positive correlation and red represents negative correlation. The correlations were done using 6-month concentration changes in each subspecies. PC: Phosphatidylcholine

Supplementary Figure 6: Correlation heatmaps of CSF and plasma TG lipid change

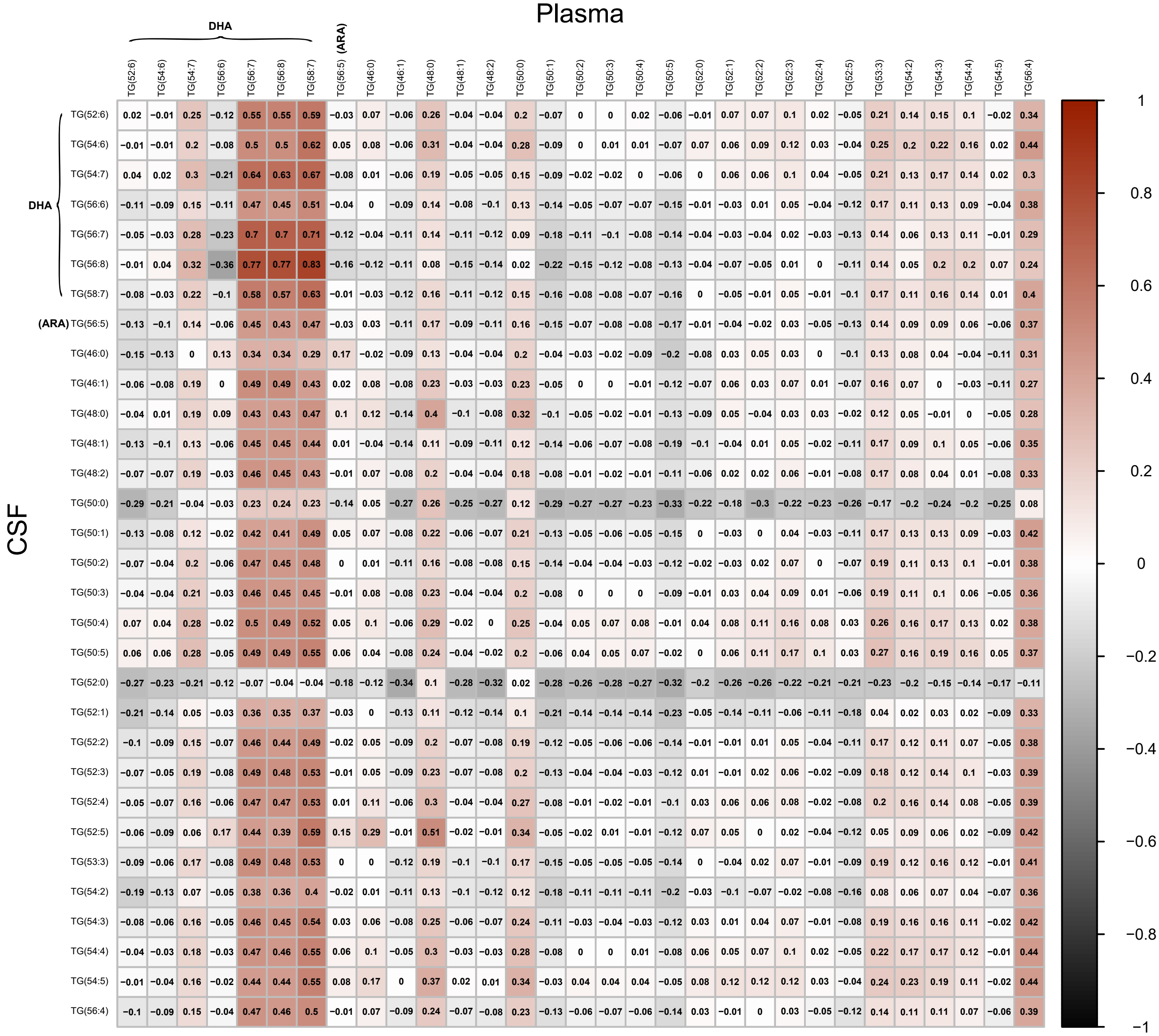

Supplementary Figure 6: Correlation heat maps between CSF and plasma lipid pools showing Pearson's correlation coefficient in each cell. Blue represents positive correlation and red represents negative correlation. The correlations were done using 6-month concentration changes in each subspecies. TG: Triglycerides

Supplementary Figure 7: HDL particles containing ARA-lipids

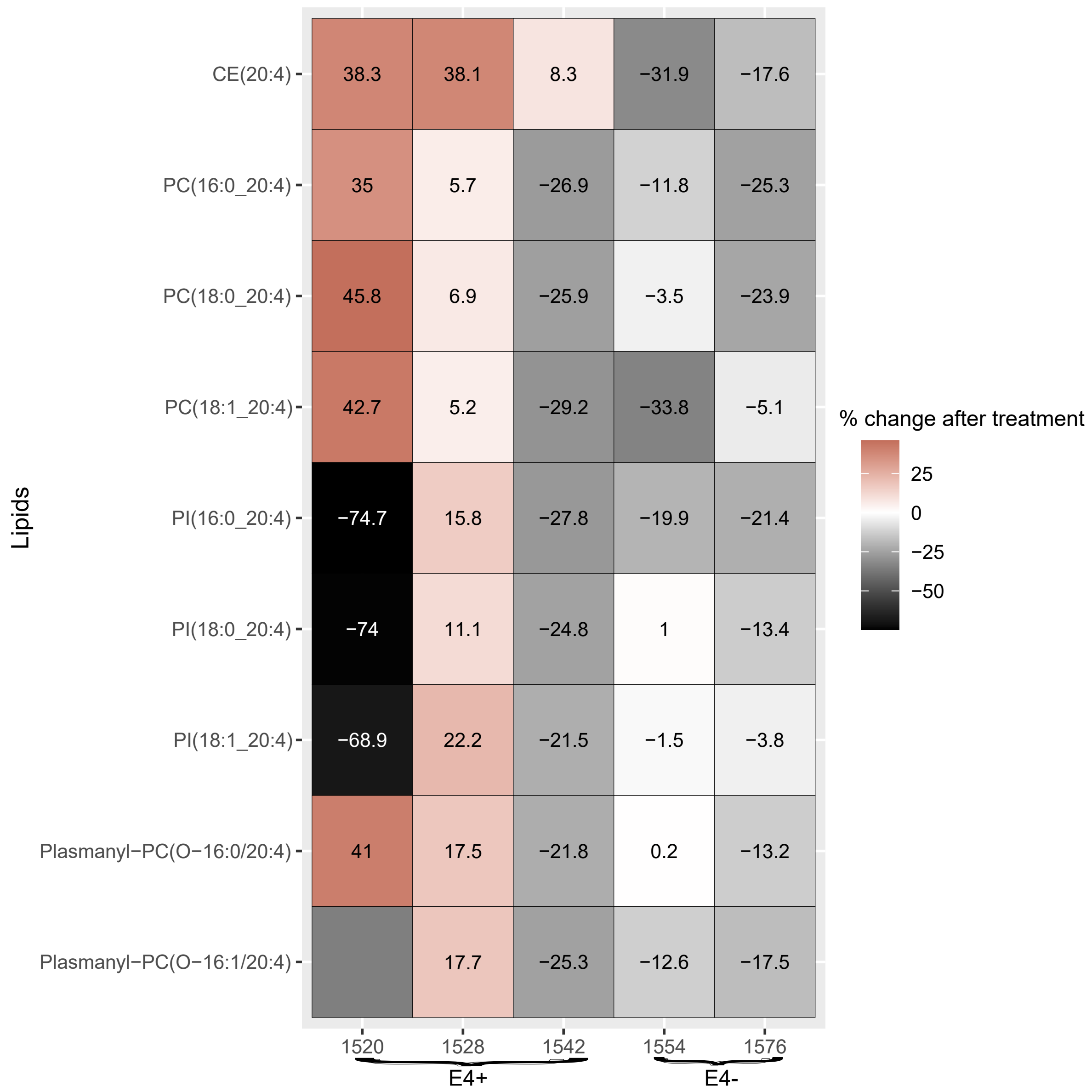

Supplementary Figure 7: HDL particle concentrations were measured in five subjects and the percentage change in ARA-containing lipids after treatment was compared between APOE4-carriers and non-carriers.
